# A scoping review of web-based, interactive, personalized decision-making tools available to support breast cancer treatment and survivorship care

**DOI:** 10.1007/s11764-024-01567-6

**Published:** 2024-03-28

**Authors:** Kaitlyn M. Wojcik, Dalya Kamil, Julia Zhang, Oliver W. A. Wilson, Laney Smith, Gisela Butera, Claudine Isaacs, Allison Kurian, Jinani Jayasekera

**Affiliations:** 1 Health Equity and Decision Sciences Research Laboratory, Division of Intramural Research, National Institute On Minority Health and Health Disparities, National Institutes of Health, Bethesda, MD 20892, USA; 2 Williams College, Williamstown, MA, USA; 3 Frederick P. Whiddon College of Medicine, Mobile, AL, USA; 4 Office of Research Services, National Institutes of Health Library, Bethesda, MD, USA; 5 Georgetown University Medical Center and Cancer Prevention and Control Program, Georgetown-Lombardi Comprehensive Cancer Center, Washington, DC, USA; 6 Departments of Medicine and Epidemiology and Population Health at Stanford University School of Medicine, Stanford, CA, USA

**Keywords:** Breast cancer, Decision-making tools, Treatment, Survivorship

## Abstract

**Purpose:**

We reviewed existing personalized, web-based, interactive decision-making tools available to guide breast cancer treatment and survivorship care decisions in clinical settings.

**Methods:**

The study was conducted using the Preferred Reporting Items for Systematic reviews and Meta-Analyses extension for Scoping Reviews (PRISMA-ScR). We searched PubMed and related databases for interactive web-based decision-making tools developed to support breast cancer treatment and survivorship care from 2013 to 2023. Information on each tool’s purpose, target population, data sources, individual and contextual characteristics, outcomes, validation, and usability testing were extracted. We completed a quality assessment for each tool using the International Patient Decision Aid Standard (IPDAS) instrument.

**Results:**

We found 54 tools providing personalized breast cancer outcomes (e.g., recurrence) and treatment recommendations (e.g., chemotherapy) based on individual clinical (e.g., stage), genomic (e.g., 21-gene-recurrence score), behavioral (e.g., smoking), and contextual (e.g., insurance) characteristics. Forty-five tools were validated, and nine had undergone usability testing. However, validation and usability testing included mostly White, educated, and/or insured individuals. The average quality assessment score of the tools was 16 (range: 6–46; potential maximum: 63).

**Conclusions:**

There was wide variation in the characteristics, quality, validity, and usability of the tools. Future studies should consider diverse populations for tool development and testing.

**Implications for cancer survivors:**

There are tools available to support personalized breast cancer treatment and survivorship care decisions in clinical settings. It is important for both cancer survivors and physicians to carefully consider the quality, validity, and usability of these tools before using them to guide care decisions.

## Introduction

Breast oncologists and surgeons have long recognized that breast cancer care should be refined by individual patient needs, preferences, and values, as patients may respond to treatment differently based on a variety of factors. Over the last three decades, personalized care has gained traction with the emergence of genomic medicine [1], ‘big data’ [2], digital health [3, 4], and advanced treatment for breast cancer [5, 6]. In this context, several web-based, interactive decision-making tools have been introduced to clinical practice to support personalized breast cancer care [7–11]. These breast cancer-specific tools were designed to provide tailored outcomes and care recommendations considering individual demographic (e.g., age) [12], genomic (e.g., 21-gene recurrence score) [13], clinical (e.g., tumor size) [14], behavioral (e.g., smoking) [15], and contextual (e.g., insurance status) [16] characteristics together with patient needs, preferences, and values [17]. For example, the ‘BreastCHOICE’ tool is a personalized decision-making tool used to estimate the risk of surgical complications in early-stage breast cancer patients considering breast reconstruction based on their individual height, weight, past medical history, smoking status, and personal preferences/values [15].

Overall, studies have shown that personalized decision-making tools could increase knowledge, reduce negative emotions, such as anxiety and fear, associated with treatment, and improve overall quality of life among breast cancer patients and survivors [7, 18–20]. Furthermore, breast cancer decision-making tools that include contextual factors, such as treatment costs, insurance status, and access to treatment facilities, could potentially help address root causes of disparities in clinical settings [21–24]. For example, decision-making tools for medical situations, including chest pain, diabetes, Graves’ disease, depression, osteoporosis, and cardiovascular risk prevention, have shown that tools that raise cost as an issue could increase the occurrence of conversations related to the costs of drugs, insurance, and health care between patients and their physicians [25].

Recently, the U.S. Food and Drug Administration (FDA) issued a guidance to regulate decision-making tools as medical devices, increasing the focus on using high-quality tools to support clinical care in the U.S. [26]. However, there are several barriers to integrating high-quality personalized decision-making tools into current clinical care [19]. For instance, physicians and patients have reported a lack of understanding of existing tools, limited knowledge on how these tools can be used to support clinical care, and as a result, low motivation to use decision-making tools to guide clinical care [27–29]. Studies have also found that both patients and physicians have limited knowledge on the validity, usability, and quality of existing tools to assess their performance in real-world practice settings [30–34].

While breast cancer decision-making tools exist, there is limited information about their quality, validity, usability, feasibility, and acceptability. We aimed to fill this knowledge gap by critically reviewing the characteristics of existing English-language, interactive, web-based personalized decision-making tools available to support breast cancer care. The overarching goal of our review was to present evidence on the existing decision-making tools for breast cancer treatment and survivorship to support the integration of these tools into clinical practice.

## Methods

This scoping review followed the methodological framework initially proposed by Arksey and O’Malley, Levac and colleagues, and the Joanna Briggs Institute [35–37]. This framework includes six stages to guide scoping review processes: (1) specifying the research question, (2) identifying relevant literature, (3) selecting studies, (4) data mapping, (5) summarizing, synthesizing, and reporting the results, and (6) including expert consultation. Our review was conducted in accordance with the Preferred Reporting Items for Systematic reviews and Meta-Analyses extension for Scoping Reviews (PRISMA-ScR) Checklist (Supplemental Table 1) [38]. The study was registered in Open Science Framework [39]. Since the study included a review of published articles and study-level results, institutional review board approval or exemption was not required.

### Data sources and search strategy

We conducted a search of published literature to identify articles that discussed personalized, interactive, dynamic, web-based decision-making tools designed to support breast cancer treatment and survivorship decisions for physicians and individuals diagnosed with breast cancer. The comprehensive search strategy included a combination of keywords, synonyms, Medical Subject Headings (MeSH), and Emtree terms relating to concepts of clinical decision-making tools, survivorship, treatment, web-based, personalized, and breast cancer (Supplemental Table 2). A trained librarian (GB) at the National Institutes of Health pilot tested 50 articles and refined our search strategy based on the initial search results. We searched PubMed, PsycInfo, Embase, Scopus, Web of Science, and Cochrane Database of Systematic Reviews for relevant articles. After screening all the articles from the database searches, we reviewed the reference lists of the articles to identify any additional tools that may have been missed, and these additional relevant articles were screened based on inclusion/exclusion criteria. The date of our most recent search was May 12, 2023.

### Inclusion and exclusion criteria

For all articles, the inclusion criteria included: (1) female or male adults (≥ 18 years) diagnosed with breast cancer, (2) breast cancer treatment or survivorship, (3) online, web-based risk prediction models and interactive, personalized, or individualized tools developed from 2013 to 2023, (4) primary empirical research studies, and (5) articles written in English. We limited our search to include tools from 2013 to 2023, as these tools are more likely to consider the most up-to-date information on breast cancer treatment and survivorship care. Additional information is provided in Supplemental Table 3.

### Data screening, extraction, and assessment of articles and tools

All titles and abstracts from articles retrieved from the databases were initially screened for eligibility by four authors (KW, DK, JZ, LS) based on the inclusion and exclusion criteria. A second round of screening using the same criteria was conducted via a full text review of the remaining articles. Screening was done using Covidence, an online application that helps streamline the review process [40]. Disagreements between authors were resolved through discussions.

We visited each tool’s publicly available website and tested each tool with pseudo patient characteristics to identify patient inputs used for personalization and breast cancer outcomes included in the tool. For tools that did not have publicly available websites, we reviewed screenshots and examined the tool development section in the methods of each corresponding article to retrieve information. We contacted the corresponding author for missing information. We used the articles, websites, and relevant screenshots to extract information about each tool, including the name and purpose, target population for tool development, interventions, data source and methods, input factors (e.g., individual, clinical, genomic, behavioral, contextual) used for personalization, breast cancer outcome/s, target user/s, and date of last update.

We also reviewed articles that provided information on tool validation, usability, feasibility, and acceptability testing. Personalized, web-based decision-making tools typically use statistical and/or simulation models to estimate outcomes associated with various input factors. After model development, these models are validated in independent, external samples to evaluate model performance and generalizability [41]. Usability testing is designed to capture the user experience and understanding of the tool, while feasibility testing helps infer the likelihood that the decision-making tool will be used to enhance the patient-physician interaction [32–34]. Acceptability testing is conducted to evaluate user satisfaction with the tool [32–34]. We extracted information on the distribution of race and ethnicity, education, income, marital status, and insurance in the sample of individuals included in validation, usability, feasibility, and acceptability testing of the tools. Data were extracted using Covidence and Excel [40].

### Quality assessment

We used the International Patient Decision Aid Standard (IPDAS) instrument to assess the quality of each tool included in our study [42]. The IPDAS collaboration considers a decision aid to be any tool that helps people make decisions about health care [43]. The IPDAS instrument was selected for the quality assessment since it was established to provide a standardized framework and a set of criteria to evaluate the content, development, and implementation of decision tools used to support health care decisions [43]. These criteria may be useful to a wide range of individuals who may use decision tools such as patients, healthcare providers, tool developers, researchers, and policymakers [42, 43].

Accordingly, the IPDAS instrument checklist evaluates tools based on the presentation of information, ability to clarify patient values, tool development process, story usage, the impact of the tool on decision processes, and decision quality [42, 43]. The full IPDAS instrument checklist is accessible in Supplemental Table 4. In our study, the tools were scored from a range of 0 to 63, with increasing scores representing the increasing number of items from the IPDAS instrument checklist represented in each tool. Finally, we summarized the overall strengths and weaknesses of each tool considering the IPDAS instrument checklist [42].

## Results

### Search results

**Fig. 1:**
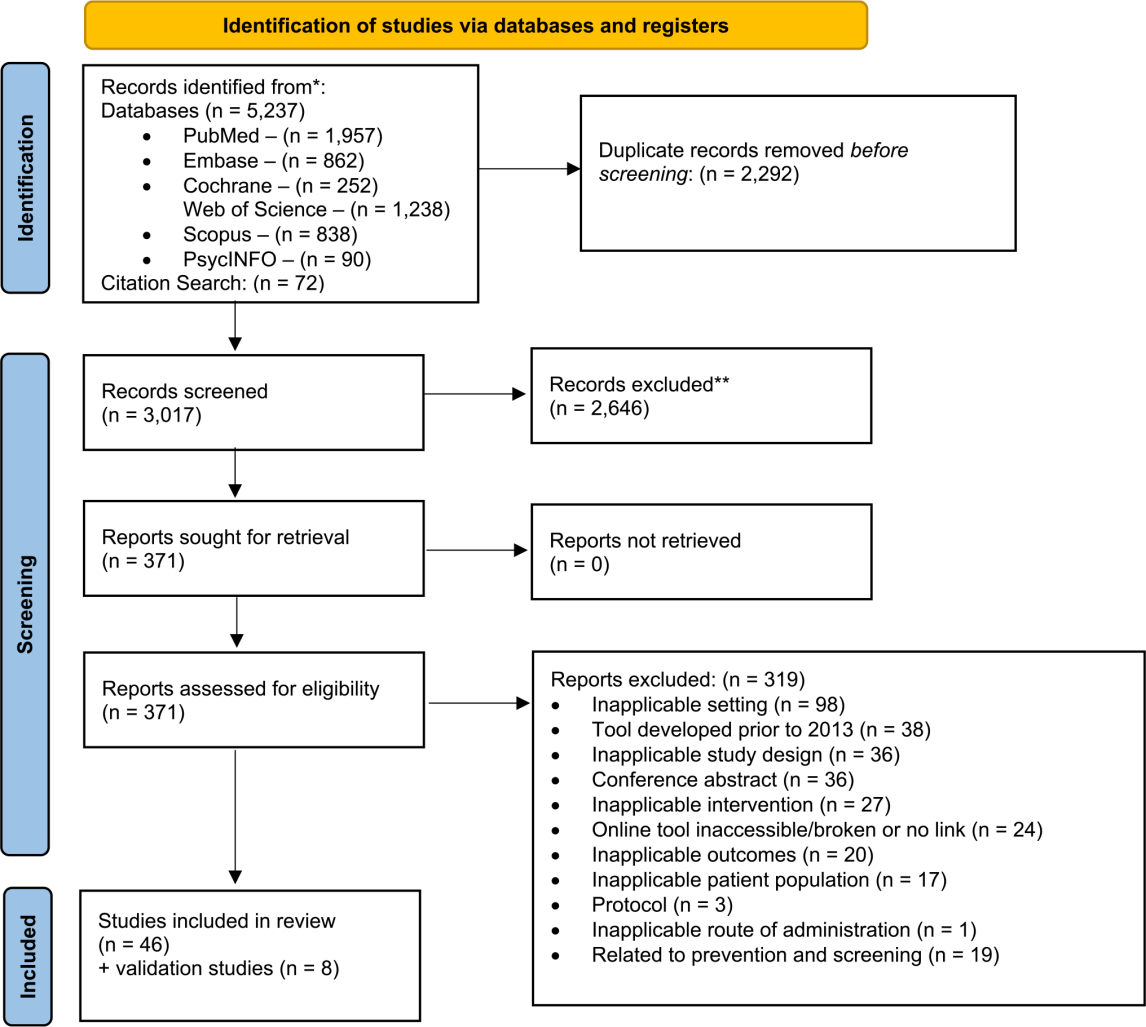
Article identification process using research framework. From: Page MJ, McKenzie JE, Bossuyt PM, Boutron I, Hoffmann TC, Mulrow CD, et al. The PRISMA 2020 statement: an updated guideline for reporting systematic reviews. BMJ 2021;372:n71. https://doi.org/10.1136/bmj.n71

A total of 5,237 records were identified through PubMed, PsycInfo, Embase, Scopus, Web of Science, and Cochrane Database of Systematic Reviews. After removing duplicates, irrelevant, and ineligible articles, a total of 46 relevant articles were included in this study (Fig. 1). These articles described 54 tools, including 11 tools that provided personalized breast cancer treatment outcomes based on individual factors (e.g., age, tumor characteristics). The remaining 43 tools provided breast cancer outcomes associated with individual factors but did not include treatment-specific personalized breast cancer outcomes.

### Personalized tools for treatment outcomes (*N* = 11)

These tools varied by target population, inputs, outcomes, and treatment considerations (Table 1) [13–15, 17, 44–49]. The tools were developed for adult women (≥ 18 years) with early-stage breast cancer [13–15, 17, 44, 45, 47–49] or ductal carcinoma in situ (DCIS) [46] considering different types of treatment. The target users for two tools were only patients [15, 17], while four tools were developed for physicians only [47–49], and nine tools were developed for both physicians and patients [13, 14, 44–46]. Two tools were developed specifically for older women [45, 49]. Four tools predicted treatment outcomes for local–regional or distant recurrence risk [13, 14, 44, 45], and five tools predicted breast cancer mortality [44–46, 49], while the other tools predicted other treatment outcomes. Several tools (*N* = 4) included multiple outcomes [14, 44–46]. For example, ‘BTxChoice’ provided estimates for the 10-year risk of distant recurrence and life-years gained with and without chemotherapy treatment [14].

The tools varied by inputs used to estimate breast cancer treatment outcomes. All tools included individual and clinical characteristics, such as age and tumor size. Two tools considered genomic features measured by the 21-gene recurrence score [13, 14], and two tools considered health behaviors [15, 45]. No tools considered the impact of contextual factors, such as insurance status or access to a treatment facility. One tool helped elicit patient preferences and values by providing a brief survey outlining patients’ thoughts and feelings about treatment options [15]. We found one tool considering the variation of breast cancer outcomes based on race and ethnicity [46].

### Validation, usability, feasibility, and acceptability testing

Six tools were externally validated [13–15, 46–48], three tools were internally validated [13, 14, 44], and three tools did not undergo any validation [17, 45, 49]. Five tools provided results from usability, feasibility, and/or acceptability testing [14, 15, 17, 45, 46]. ‘BreastCHOICE’ had a high mean usability score of 6.3, which was measured using the Computer System Usability Questionnaire, providing a score ranging from 1.0 (lowest) to 7.0 (highest) [15, 50]. ‘Which treatment for DCIS is right for you?’ had a mean usability score of 3.7 out of 5.0 measured using the System Usability Scale and the Preparation for Decision-Making Scale [46, 51, 52]. ‘BTxChoice’ and ‘Radiotherapy for Older Women’ did not report results from usability testing, but the authors stated that the tools were in the process of undergoing testing [14, 45]. ‘BRECONDA’ underwent acceptability and feasibility testing; it was assessed for usefulness and relevancy on a Likert-scale from 1 (lowest) to 5 (highest), with the tool receiving mean scores of 4.8 and 4.4, respectively [17]. Follow-up studies confirmed acceptability of the tool [53, 54].

Supplemental Table 5 provides the distribution of race and ethnicity, income, education, marital status, and insurance status of the individuals included in the validation and usability testing of the tools. Most patients included in validation and usability testing were White (68.2–83.9%) and married (71.1–86.0%).

### Personalized tools for other outcomes (*N* = 43)

A total of 43 tools included models to estimate breast cancer outcomes associated with individual, tumor, and contextual characteristics, but did not include treatment-specific personalized breast cancer outcomes (Table 2) [16, 55–87]. These tools were created for adult (≥ 18 years) female and/or male breast cancer patients who had undergone treatment for DCIS or invasive breast cancer. Ten tools were developed for patients with bone or lung metastases after a breast cancer diagnosis [56, 57, 69, 73, 75]. Four tools were created for young breast cancer patients (18–40 years) [16, 68, 78], and another three were created for elderly patients (≥ 65 years) [64, 65, 74]. Three tools were developed specifically for male breast cancer patients with bone metastases [73]. The target user for four tools was patients [55, 78, 80, 81], while 31 were developed only for physicians [16, 56–59, 62–65, 67–71, 74–77, 79, 82–84, 86, 87], and eight were developed for both physicians and patients [60, 61, 66, 72, 73, 85]. The most common outcomes estimated in these tools included overall survival (*N* = 20) [16, 57, 62–64, 66–77, 79, 85, 86], breast-cancer specific survival (*N* = 7) [16, 57, 65, 70, 73, 75, 79], and risk of bone metastasis (*N* = 3) [56, 69, 73]. The ‘After Cancer Education and Support Operations’ tool was the only tool developed to support breast cancer survivors by providing health alerts and follow-up care recommendations after treatment [55].

All tools considered individual and clinical factors such as age and tumor stage [16, 55–87]. Four tools considered health behaviors, such as smoking status [59, 60, 78, 82]. Twenty-one tools incorporated contextual factors, including marital status (*N* = 20) [16, 57, 62–65, 69, 70, 72–76, 79], insurance status (*N* = 4) [16, 69], education (*N* = 1) [78], employment status (*N* = 1) [78], and financial status (*N* = 1) [78]. Only two tools included components to incorporate patient preferences or values into decision-making [78, 80]. We found 17 tools considering Black, White, and other race categories to estimate breast cancer outcomes [16, 56, 57, 59, 62–65, 69, 70, 75, 76]. One tool considered Hispanic and non-Hispanic ethnicities [59].

### Validation, usability, feasibility, and acceptability testing

We found that 15 tools were externally validated [55, 56, 58, 59, 61, 65, 68, 69, 71, 76, 79, 84], and 35 tools were internally validated [16, 56, 57, 59, 60, 62–77, 79, 83, 84, 86, 87]. Five tools did not undergo any validation testing [78, 80–82, 85]. Only four tools underwent usability, feasibility, and/or acceptability testing [55, 78, 80, 81]. ‘After Cancer Education and Support Operations’ assessed usability and acceptability using ‘Consistency’, ‘Stressfulness’, and ‘Simplicity’ with scores ranging from 1 (most positive) to 7 (most negative) [55, 88]. Consistency refers to the ability to use the tool in the same way over time, while stressfulness refers to the amount of worry or tension caused by the use of the tool, and simplicity refers to the ease of use of the tool [55, 89]. Users reported a mean consistency score of 1.2, a mean stressfulness score of 1.2, and a mean simplicity score of 1.4 for this tool [55]. The tool developed by Graetz et al. was tested for feasibility based on reports from physicians and nurses who used it; providers stated that the tool was easy to implement and did not significantly impact workflow [81]. The ‘Resources and Education for Adherence to Cancer Hormonal Therapy’ tool was assessed for feasibility and acceptability [80], where the study aimed to have 80% of eligible individuals enroll with 80% completing at least one online session. Both goals were exceeded for this tool, with 85.4% of eligible individuals enrolling and 83.7% of individuals completing at least one session [80]. Acceptability was measured using the ‘Client Satisfaction Questionnaire’ and the ‘Intervention Feedback Questionnaire’ [90]. The ‘Resources and Education for Adherence to Cancer Hormonal Therapy’ tool had a mean acceptability score of 3.0 (range 1–4) and 3.4 (range 1–5) on both questionnaires, respectively [80].

Supplemental Table 5 provides the distribution of race and ethnicity, income, education, marital status, and insurance status of the individuals included in validation, usability, feasibility, and acceptability testing of these tools. Most patients were White (0–93.0%), married (41.4–94.0%), and had insurance (93.7–94.9%).

### Quality assessment

The sum of the scores for each tool in each dimension on the IPDAS instrument checklist is reported in Supplemental Table 6. The tools could receive scores ranging from 0 (lowest quality) to 63 (highest quality). Most tools provided information about options (*N* = 48) and outcome probabilities (*N* = 48), were written in plain language (*N* = 49), and were easy to navigate online (*N* = 51). However, only six tools provided disclosure information about funding or conflicts of interest, and only two tools used stories. In our sample, the average quality assessment score for the tools was 16 (range: 6–46; potential maximum: 63). The tool with the highest IPDAS instrument score was ‘BreastCHOICE’, with 46 points. ‘BreastCHOICE’ provided information on different options and the development process while also sufficiently incorporating patient values and preferences into the decision-making tool by asking patients what matters most to them, what their concerns were, and how they feel about different treatments [15].

### Summary: strengths and weaknesses

We provided a list of strengths and weaknesses of the web-based decision-making tools included in our study in Table 3. In terms of strengths, we found that most tools were written in plain language (*N* = 49), were validated (*N* = 45), and provided information about breast cancer outcomes (*N* = 48). However, usability, feasibility, and acceptability of the tools were evaluated using different measures. As a result, it was not possible to compare the performance of the tools. There was also limited information on the validity and usability testing of the tools in underserved (e.g., uninsured, low education) and underrepresented (e.g., Alaska Native, Pacific Islanders) populations.

## Discussion

Breast cancer care decisions are complex and often require the consideration of individual, clinical, genetic, health behavioral, and contextual characteristics, as well as personal preferences and values, to achieve optimal treatment outcomes. In this scoping review, we identified 54 web-based, personalized, interactive decision-making tools that could be used to support breast cancer care in clinical settings.

### Comparison with other literature

Previous studies have reviewed up to 21 tools, including risk prediction models, to support breast cancer treatment decisions [7, 91, 92]. In contrast, we identified a broader set of tools that could potentially be useful to support breast cancer treatment and survivorship care decisions in clinical settings. Like previous reviews, we also found that most tools still need to undergo usability, feasibility, and acceptability testing [7, 91, 92]. However, in this study, in addition to an appraisal of tool validity, usability, feasibility, and acceptability, we also evaluated the inclusion of underrepresented and underserved populations in tool development and testing. We found that individuals included in post-testing of the tools were mostly White, insured, married, and had higher levels of education. Moreover, previous reviews have provided limited information on health behaviors and contextual factors that may also influence breast cancer outcomes [7, 91, 92]. To our knowledge, this is the first to provide a detailed and comprehensive evaluation of the web-based decision tools considering health behaviors, contextual factors, and the characteristics of the populations included in validity and usability testing of these tools.

### Summary of main findings

Tool validation is a necessary step in decision-making tool development, as it provides critical information on the tools’ ability to accurately estimate outcomes of interest in independent cohorts [93]. A tool’s performance (e.g., sensitivity, specificity) may vary based on the distribution of individual, clinical, and contextual characteristics of a given cohort [94]. Therefore, it is important to test the external validity of the decision-making tools (and related algorithms) in independent cohorts prior to the introduction of these tools into practice settings. Validation could also help identify additional important features that may have been missed in the initial development of the tool, which could help further increase the accuracy of the prediction. The validation samples for the tools in our review included mostly White, married, and insured populations. For example, ‘BTxChoice’ was validated in two populations, both with a White majority (73.0–83.9%) [14]. These findings were consistent with previous studies reporting that only 14% of decision tools were tested with a significant representation of underserved and underrepresented groups [95]. The lack of representation in validation samples could limit the ability to assess the performance of these tools in diverse settings [95]. Importantly, if the tools are unable to generate accurate estimates for certain subgroups of the population, using them to guide clinical decisions could perpetuate disparities in cancer care and outcomes. Therefore, it is necessary to develop and validate tools in diverse cohorts including underserved and underrepresented individuals.

Usability testing is a necessary step in tool development to help identify and fix problems with the website/mobile application, [96] but few tools in our review had undergone usability testing. During usability testing, tool developers should assess the tools’ ease of use and the presentation of information considering health literacy and numeracy [97, 98]. Studies have shown that tools that are difficult to use are often neglected despite their utility [99]. Usability testing that includes individuals with different levels of health literacy and numeracy could potentially enhance the long-term utility of these tools in clinical settings [96–98].

Several tools considered health behaviors, such as smoking status and alcohol intake. Health behaviors are important predictors of breast cancer mortality and survivorship [100]. While physical activity was not considered a health behavior in most of the decision-making tools included in our study [78], previous studies have shown that increased physical activity could lower breast cancer recurrence and mortality [101, 102]. Current smoking, dietary intake, sedentary behavior, and poor sleep are also known to be associated with breast cancer mortality [103–105]. Inclusion of these factors in breast cancer decision-making tools could potentially help patients identify resources (e.g., smoking cessation interventions for quitting) to improve behavior and help physicians develop survivorship care plans considering these factors.

Few tools considered patients’ preferences and values by asking patients their thoughts and concerns about different treatments and what matters most to them. Patients may have a wide range of preferences and values when considering the benefits and harms of treatment. Patients who receive their preferred treatment have been shown to be half as likely to stop treatment, and patients who are actively involved in decision making throughout their cancer care by voicing their preferences and values report a higher quality of life [18, 106]. Additionally, tools that incorporate patient values, such as cultural values, spirituality, and community, often improve the communication between patients and physicians, leading to improved shared decision making [107].

The debate over whether to include race and ethnicity in risk prediction models is ongoing, and not many tools included race or ethnicity as input variables. Race-based medicine has been used to deliver healthcare for years based on epigenetics, but it has a deeply problematic history used to reinforce and justify slavery and perpetuate racial discrimination [108]. Furthermore, racial categories change over time, which may mean that older tools that have not been updated may not be as relevant or accurate [108]. Currently, there is a push to consider race as an input factor only when it is directly connected to racism and contextual factors [109]. Studies have shown that contextual factors such as lack of health insurance, income, food insecurity, and access to treatment facilities contribute to the racial and ethnic disparities in breast cancer mortality [110, 111]. Therefore, the consideration of these factors in decision-making tools could potentially provide a means to reduce racial and ethnic disparities in breast cancer outcomes in the U.S. [112].

Less than half of the decision-making tools personalized breast cancer outcomes based on individual contextual factors such as insurance, education, employment, marital status, and financial status/burden. We considered marital status as a contextual factor due to the marriage protection theory [113], which posits that marriage may lead to improved breast cancer survival through the strengthening of interpersonal relationships, providing social and financial support, and reducing risky behaviors [114, 115]. Studies have also shown that living in highly segregated neighborhoods in the U.S. are associated with lower rates of breast cancer survival [116, 117]. The inclusion of these factors in decision-making tools may provide an opportunity for physicians to discuss, advocate, and ensure that patients’ full range of circumstances are accounted for when making informed decisions about breast cancer care.

### Strengths and limitations

Our review has several limitations that should be considered when evaluating our findings. We did not consider web tools created prior to 2013 or in any language other than English because we wanted to limit our review to include the most recent, relevant tools. However, this means that our search likely did not encompass the full range of personalized decision-making web tools that are currently available for breast cancer care. Additionally, we only assessed tools that were developed in the U.S., Europe, Australia, Japan, and Korea. Because of this, tools may not be generalizable or applicable to all populations. We were unable to access 23 tools due to payment barriers or because only screenshots with incomplete information were available in the publications. As a result, we were unable to assess the quality of all the components of those tools that were not easily accessible. Also, we were unable to report the characteristics of the samples included in the validation, usability, feasibility, and acceptability testing of 18 tools, as this information was not readily available in the original studies.

Despite these limitations, we conducted a robust search for personalized web-based clinical tools and identified a significant number of tools that assessed breast cancer treatment and survivorship outcomes. To our knowledge, this is the first scoping review providing a detailed assessment and comparison of the web-based decision tools available to support breast cancer care in clinical settings.

## Conclusions

There was wide variation in the characteristics, validity, usability, and quality of web-based, interactive decision-making tools available to support breast cancer care. We found that the quality assessment tool (i.e., the IPDAS instrument checklist) did not include components to evaluate contextual factors which may influence patient decisions, the ability to seek health care, and patient outcomes [42]. The inclusion of contextual factors in the IPDAS instrument checklist could motivate tool developers to include these factors in new decision-making tools.

We expect the quality and the use of these tools to increase with the new U.S. FDA regulation [26]. However, it is important to concurrently provide training to patients and physicians to ensure that these tools are used for their intended purposes [27–29, 118]. Further, integrating decision tools into electronic medical records systems could improve clinical workflow, the speed and quality of decision making, and communication between physicians and their patients [119].

## Supplementary Material

Supplement

## Figures and Tables

**Table 1:** Web-based decision-making tools for personalized treatment outcomes (*N* = 11)

Tool name	Purpose	Target population	Intervention	Data source	Methods used to estimate outcomes	Inputs							Outcome(s)	Target user/s	Validation	Usability/feasibility/acceptability testing	IPDAS instrument score (range: 0–63)

						Individual, clinical, treatment	Other treatment received	Genomic	Health behaviors	Contextual	Race and ethnicity	Preferences and values					

Age Gap Decision Tool—1 [49, 120]	To allow older women to compare breast cancer treatments	Women 70+ with primary operative early invasive breast cancer	Surgery plus adjuvant endocrine therapy vs. primary endocrine therapy	46 breast units in England and Wales	Generalized linear model, Cox proportional hazards regression	Age, comorbidities, frailty, nodes positive, tumor grade, tumor size	-	-	-	-	-	-	2- and 5-year survival	Physicians	-	-	32
Age Gap Decision Tool—2 [49, 121]	To allow older women to compare breast cancer treatments	Women 70+ with primary operative early invasive breast cancer	Whether to have adjuvant chemotherapy after surgery	46 breast units in England and Wales	Generalized linear model, Cox proportional hazards regression	Age, comorbidities, ER status, frailty, HER2 status, nodes positive, tumor grade, tumor size	-	-	-	-	-	-	2- and 5-year survival	Physicians	-	-	32
BreastCHOICE [15] (Limited availability; accessible through Principal Investigator)	To educate patients about breast reconstruction, estimate risk of complications, and clarify patient preferences	Adult females with stage 0–3 breast cancer considering post-mastectomy breast cancer reconstruction with no previous reconstruction attempts	Breast reconstruction (implant, flap, immediate, delayed)	HealthCore Integrated Research Database	Multivariable generalized linear models	Height, past medical history, weight	Radiotherapy	-	Smoking status	-	-	Individual prefer ences/values regarding breast reconstruction	Complication risk	Patients	External [122]	Yes [15]	46
BRECONDA [17, 123]	To help patients make a decision about breast reconstruction	Women diagnosed with invasive breast cancer or DCIS, eligible for breast reconstruction, English literate, over 18 years, and had computer access	Breast reconstruction	Westmead Breast Cancer Institute	Tailored information based on patient’s individual preferences	-	-	-	-	-	-	Individual preferences/values regarding breast reconstruction and type of reconstruction	Preference for breast reconstruction and type of reconstruction	Patients	-	Yes [17, 53, 54]	34
BTxChoice [14] (Limited availability; accessible through Principal Investigator)	To provide breast cancer treatment prognoses and predict chemotherapy benefit	Females (40–74 years) with node-negative, hormone receptor-positive, HER2-negative, invasive breast cancer who have received lumpectomy with radiotherapy or mastectomy	Endocrine therapy, chemoendocrine therapy	Simulation modeling using existing meta-analysis, clinical trial, and observational data	Bayesian analysis	Age at diagnosis, comorbidities, tumor grade, tumor size	-	21 gene recurrence score	-	-	-	-	1. 10-year risk of distant recurrence 2. Life-years gained with vs. without chemotherapy 3. Predicted 21-gene recurrence score (if not known)	Physicians and patients	Internal, External [14]	Yes [14]	14
LinfoNeo [47, 124]	To develop a nomogram to select patients with a low risk of residual axillary disease after NAC, in which axillary surgery could be minimized	Patients with node-positive breast cancer with NAC considering axillary surgery	Axillary surgery	11 tertiary breast units coordinated by the Breast Unit of mccs Maugeri Hospital of Pavia, Italy	Multivariate logistic regression	Biomolecular subtype at core biopsy, Ki 67 at core biopsy, histological type at core biopsy, NAC regimen, post-NAC breast complete clinical response, post-NAC clinical/radiological axillary re-staging, pretreatment clinical T stage	-	-	-	-	-	-	Likelihood of nodal pCR after NAC	Physicians	External [47]	-	18
Meretoja et al. 2017 [48] (Limited availability; Accessible through screenshots in paper)	To develop a tool to predict the risk of persistent pain after breast cancer surgery	Patients with unilateral breast cancer undergoing surgery	Surgery	Breast cancer units at the Helsinki University Hospital, units in Northern Scotland, and the Rigshospitalet	Binary logistic backward stepwise regression	Axillary operation, BMI, first postoperative day acute pain, seventh day postoperative acute pain	-	-	-	-	-	-	Probability of moderate to severe persistent pain	Physicians	External [48]	-	11
Outcomes Predictor after Mastectomy with N1 Breast Cancer [44, 125]	To provide objective, personalized risk estimates of outcomes of post-mastectomy radiation therapy	Female patients with invasive breast cancer treated with mastectomy, tumor size 5 cm or less, 1–3 positive lymph nodes, and no evidence of metastatic disease	PMRT	Five North American institutions	Competing risks regres sion	Age at diagnosis, ER/PR status, grade, HER2 status, lymphovascular invasion, number of lymph nodes sampled, number of positive lymph nodes, pathologic tumor size, tumor location (inner or other)	Optimal systemic therapy (receiving endocrine therapy if ER or PR positive, trastuzumab if HER2 positive, and chemotherapy if ER and PR negative)	-	-	-	-	-	1. 5- and 10-year local recurrence with and without PMRT 2. 5- and 10-year distant recurrence with and without PMRT 3. 5- and 10-year any recurrence with and without PMR 4. 5- and 10-year breast cancer mortality with and without PMRT	Physicians and patients	Internal [44]	-	21
Radiotherapy for Older Women [45, 126]	To calculate risk of local recurrence and survival for older women with early-stage breast cancer	Older adult women (65 years+) deciding whether to undergo radiotherapy for early stage, ER positive breast cancer who have undergone breast conserving surgery	Radiotherapy	Advisory committee consisting of researchers, advocates, clinicians, and patients	Markov simulation model	Age, chronic lung disease, congestive heart failure, diabetes or high blood pressure, functional status, height, previous history of cancer, weight	-	-	Difficulty managing money, smoking status	-	-	-	1. Local recurrence 2. 10-year overall survival	Physicians and patients	-	Yes [45]	26
RS Clin [13] (Limited availability; accessible through Principal Investigator)	To predict the risk of distant recurrence and chemotherapy benefit	Women with hormone receptor-positive, HER2-negative, node-negative breast cancer who received a mastectomy/Lumpectomy/radiation	Endocrine therapy, chemoendocrine therapy	NSABP and TAILORx Trials [127]	Cox regression	Age at surgery, tumor grade, tumor size	Planned endocrine therapy (tamoxifen or aromatase inhibitor), radiation therapy, type of surgery	21 gene recurrence score	-	-	-	-	10-year risk of distant recurrence	Physicians and patients	Internal, External [13]	-	-^
Which treatment for DCIS is right for you? [46] (Limited availability; accessible through supplemental information in paper)	To allow patients with DCIS to understand then-risks and surgical options	DCIS patients	Active monitoring, lumpectomy, lumpectomy with radiation, mastectomy, and hormone therapy	NCDB	Logistic regression, time-to-event regression	Age at diagnosis, comorbidities, ER/PR status, nuclear grade	-	-	-	-	Black, White, Other	-	1. Risk of invasive breast cancer on initial biopsy 2. 10-year risk of invasive breast cancer 3. 10-year competing risks of death from breast cancer and causes unrelated to breast cancer	Physicians and patients	External [46]	Yes [46]	23

*BMI* body mass index, *DCIS* ductal carcinoma in situ, *ER* estrogen receptor, *HER2* human epidermal growth factor receptor 2, *Ki 67* antigen Ki 67, *NAC* neoadjuvant chemotherapy, *NCDB* National Cancer Database, *NSABP* National Surgical Adjuvant Breast and Bowel Project, *pCR* pathologic complete response, *PMRT* post-mastectomy radiation therapy, *PR* progesterone receptor, *TAILORx* Trial Assigning Individualized Options for Treatment

- = None

^ ‘RSClin’[13] was excluded; due to a paywall, authors could not accurately assess the full tool

**Table 2:** Web-based decision-making tools for other outcomes (*N* = 43)

Tool name	Purpose	Target population	Data source	Methods used to estimate outcomes	Inputs							Outcome	Target user/s	Validation	Usability/feasibility/acceptability testing	IPDAS instrument score (0–63)

					Individual, clinical	Other treatment received	Genomic	Health behaviors	Contextual	Race and ethnicity	Preferences and values					

3 Scenarios for Survival [85, 128]	To estimate and explain personalized information about life expectancy	Adults with incurable breast cancer	Participating oncologists and their patients from Australia	Simple regression methods	Expected survival time	-	-	-	-	-	-	1. Worst-case survival 2. Most likely survival 3. Best-case survival	Physicians and patients	-	-	17
ADTree Model for Axillary Lymph Node Metastasis [84, 129]	To develop a tool to estimate the probability of axillary lymph node metastasis	Patients with primary invasive breast cancer who underwent sentinel lymph node biopsy or axillary lymph node dissection without prior treatment	Tokyo Metropolitan Cancer and Infectious Disease Center, Kyoto University Hospital	Machine learning methods	Age, BMI, detection of lymph nodes, existence of calcification, existence of masses (malignant), HER2 status, histological grade, nipple discharge, skin dimpling	-	-	-	-	-	-	Probability of axillary lymph node metastasis	Physicians	Internal, External [84]	-	13
ADTree Model for Pathological Response to Neoadjuvant Therapy [84, 130]	To develop a tool to calculate the probability of pathological complete response to neoadjuvant therapy	Patients with primary invasive breast cancer who underwent sentinel lymph node biopsy or axillary lymph node dissection without prior treatment	Tokyo Metropolitan Cancer and Infectious Disease Center, Kyoto University Hospital	Machine learning methods	Architectural distortion, BMI, ER/PR status, HER2 status, menopausal status, mitotic index, presence of calcification, presence of masses (malignant), skin dimpling	-	-	-	-	-	-	Pathological response after neoadjuvant therapy	Physicians	Internal, External [84]	-	13
After Cancer Education and Support Operations (ACESO) [55] (Limited availability; Accessible through screenshots in paper)	To design and develop a personalized Web application to support breast cancer survivors after treatment	Women with a breast cancer diagnosis who completed adjuvant cancer therapy, are currently cancer free, and have no history of treatment of other cancers	Participants from the University of Wisconsin-Milwaukee	Tailored information based on patient’s individual information	Breast cancer subtype diagnosis, fatigue, medical tests completed, medications taking, mental and emotional symptoms, mental health, mood, other symptoms, physical symptoms, procedures, sexual function, sexual function symptoms, sleep quality, weight	-	-	-	-	-	-	1. Health alerts 2. Follow-up care recommendations	Patients	External [131]	Yes [55]	14
Application of Machine Learning Methods to Predict Bone Metastases in Breast Infiltrating Ductal Carcinoma Patients [56, 132]	To develop a model based on machine learning that predicts the risk of bone metastases in infiltrating ductal carcinoma patients	Male and female patients with IDC	SEER database	Multivariate logistic regression, machine learning models	Age, breast subtype (HR/HER2 status), T/N stage, sex, tumor grade	-	-	-	Marital status	American Indian/Alaska Native, Asian or Pacific Islander, Black, White	-	Risk of bone metastasis	Physicians	Internal, External [56]	-	11
Breast Cancer Lung Metastasis Cancer-Specific Survival Nomogram [57, 133]	To create a nomogram for breast cancer patients with lung metastases to quickly, accurately, and intuitively assess cancer-specific survival rates	Adult (18+) female breast cancer patients with lung metastases	SEER database	Multivariate Cox regression	Age, ER/PR status, grade, HER2 status, T stage, bone/brain/liver metastases	Chemotherapy, surgery	-	-	Marital status	Black, Other/NOS, White	-	Cancer-specific survival	Physicians	Internal [57]	-	16
Breast Cancer Lung Metastasis Overall Survival Nomogram [57, 134]	To create a nomogram for breast cancer patients with lung metastases to quickly, accurately, and intuitively assess overall survival rates	Adult (18+) female breast cancer patients with lung metastases	SEER database	Multivariate Cox regression	Age, ER/PR status, grade, HER2 status, T stage, bone/brain/liver metastases	Chemotherapy, surgery	-	-	Marital status	Black, Other/NOS, White	-	Overall survival	Physicians	Internal [57]	-	16
Breast Cancer Nomograms: Prediction for a Low-risk and a High-risk Oncotype DX Recurrence Score [58, 135]	To develop nomograms that can be used as a substitute prediction model for high-risk or low-risk Oncotype DX recurrence score results	Females (> 18 years) with invasive breast carcinoma, ER positive, HER2 negative, no regional lymph node metastasis, and tumor size between 6 and 50 mm	NCDB	Logistic regression	Age, grade, histologic type, PR status, tumor size	-	-	-	-	-	-	Probability of high or low risk Oncotype DX recurrence score	Physicians	External [58, 136]	-	14
Breast Cancer Surgery Risk Calculator [59, 137]	To estimate the risk of four types of postoperative complications for women undergoing five surgical procedures for breast cancer (partial mastectomy, oncoplastic surgery, mastectomy alone, mastectomy with implant or tissue expander reconstruction, mastectomy with autologous tissue reconstruction)	Females (18+ years) with breast cancer admitted under general or plastic surgery	National Surgical Quality Improvement Program	Logistic regression	Age, date of surgery, diabetes, diagnosis of DCIS or invasive breast cancer, functional status, height, inpatient or outpatient, medications taken, past medical history, stage 4 metastatic cancer diagnosis, surgery complications, weight, and recent unintentional weight loss	Lymph node surgery, drain or wound assist device	-	Smoking status	-	Asian/Pacific Islander, Black, Hispanic, Indian, non-Hispanic, White	-	1. Overall complication risk 2. Infectious complication risk 3. Hematologic complication risk 4. Internal organ complication risk	Physicians	Internal, External [59]	-	21
Breast Reconstruction Risk Assessment (BRA) Score—Extended Length [60, 138]	To predict the risk of postoperative complications from breast reconstruction surgery	All adult (> 18 years) patients undergoing immediate breast reconstruction (tissue expander, pedicled abdominal (TRAM) flap, latissimus flap, microvascular reconstruction, single-stage implant)	Tracking Operations and Outcomes for Plastic Surgeons database	Generalized linear modeling	American Society of Anesthesiologists physical status classification, age, comorbidities, current medication, height, number of breasts being reconstructed, surgical history, weight	Chemo therapy, radiotherapy	-	Smoking status	-	-	-	1. 30-day and 1-year overall surgical complication risk 2. 30-day and 1-year surgical site infection risk 3. 30-day and 1-year seroma risk 4. 30-day and 1-year dehiscence risk 5. 30-day and 1-year flap loss (partial or total) risk 6. 30-day and 1-year explantation risk 7. 30-day and 1-year risk of reoperation 8. Overall 30-day medical complications	Physicians and patients	Internal [60]	-	21
CBCRisk: Contralateral Breast Cancer (CBC) Risk Predictor [61, 139]	To estimate risk of contralateral breast cancer	Females (18–88) with invasive breast cancer or DCIS who were diagnosed with contralateral breast cancer	BCSC and SEER database	Multivariate logistic regression	Age at diagnosis, age at first childbirth, breast density, current age, ER status, first degree relatives with breast cancer, history of high risk preneoplasia, type of first breast cancer	Hormonal therapy	-	-	-	-	-	Contralateral breast cancer risk	Physicians and patients	External [140]	-	13
Conditional Survival Nomogram [62] (Limited availability; accessible through results section in paper)	To develop a novel conditional survival nomogram for real-time prediction of 10-year survival	Adult (18+) male and female non-metastatic triple-negative stage I–III breast cancer patients	SEER database	Multivariate Cox regression (LASSO method)	Age at diagnosis, T/N stage	Chemotherapy, radiotherapy, surgery	-	-	Marital status	Black, Other, White	-	Overall survival	Physicians	Internal [62]	-	10
Dai et al. 2018 [63] (Limited availability; accessible through results section in paper)	To construct nomograms for the outcome prediction of triple negative breast cancer patients	Female primary triple negative breast cancer patients aged 20–79 years with invasive ductal carcinoma who had surgery	SEER database	Multivariate Cox regression	Age, grade, laterality, location, number of nodes, tumor size, tumor stage	Chemotherapy, radiotherapy	-	-	Marital status	American Indian/Alaska Native, Asian or Pacific Islander, Black, White	-	1- and 3-year overall survival	Physicians	Internal [63]	-	11
Dynamic Nomogram [64, 141]	To develop a nomogram that can predict breast cancer outcomes in elderly patients	Female triple negative breast cancer patients aged 70+	SEER database	Multivariate Cox regression	Age, grade, T/N stage, tumor size	Chemotherapy, radiotherapy	-	-	-	Black, Other, White	-	Overall survival	Physicians	Internal [64]	-	18
Dynamic Nomogram [65, 142]	To develop an individualized nomogram for estimating breast cancer specific death	Females older than 65 years with stage I-III breast cancer	SEER database	Multivariate competing risks survival analysis	Age, ER/PR status, grade, histology, T/N stage	Surgery	-	-	Marital status	Black, Other, White	-	Breast-cancer specific survival	Physicians	Internal, External [65]	-	15
Dynamic Nomogram for Breast IMPC After Mastectomy [87, 143]	To develop a prognostic nomogram to estimate personalized risk of locoregional recurrence	Patients with breast IMPC who underwent mastectomy	Patients from Harbin Medical University Cancer Hospital	Multivariate Cox regression	Age, histologic grade, hormone receptor status, lymph node metastasis, lymphovascular invasion	Radiotherapy	-	-	-	-	-	Locoregional recurrence-free survival	Physicians	Internal [87]	-	11
Dynamic Nomogram for Predicting Survival of Locally Advanced Breast Cancer [66, 144]	To develop a personalized conditional survival nomogram to calculate overall survival and identify high-risk patients	Male and female locally advanced breast cancer patients (T3N0M0 and stage III disease)	SEER database	Multivariate Cox regression	Age, ER/PR status, grade, HER2 status, T/N stage	Chemotherapy, radiotherapy, surgery	-	-	-	-	-	Overall survival	Physicians and patients	Internal [66]	-	13
Equation [67, 145]	To improve prognostic predictive value and risk stratification among breast cancer patients	Adult (18+) females diagnosed with breast cancer at first malignancy	SEER database	Multivariate Cox regression	Age, ER/PR status, grade, HER2, TNM stage	-	-	-	-	-	-	Overall survival	Physicians	Internal [67]	-	10
Graetz et al. 2018 [81] (Limited availability; accessible through screenshots in paper)	To design a web-based application that allows breast cancer patients to report adherence and symptoms to increase adherence of AIs	Adult (18+) females diagnosed with early stage (0-III) HR+ breast cancer and a prescription for AI	West Cancer Center in Memphis, TN	Independent t-tests and chi-square tests	Adherence to AI, symptoms of AI use	-	-	-	-	-	-	1. Self-reported AI adherence 2. Symptom burden of AI	Patients	-	Yes[81]	-^
Huang et al. 2022 [68] (Limited availability; accessible through results section in paper)	To create a nomogram to predict overall survival in young breast cancer patients	Female patients diagnosed with young breast cancer (under 40)	SEER database	Univariate Cox regression (LASSO method)	ER/PR status, grade, HER2 status, lymph node ratio, T/N stage	-	-	-	-	-	-	3- and 5-year overall survival	Physicians	Internal, External [68]	-	6
Huang et al. 2020 [69] (Limited availability; accessible through results section in paper)	To develop a predictive model by analyzing SEER data to determine the risk of bone metastases in patients with IDC	Male and female patients with newly diagnosed IDC	SEER database	Multivariate Cox regression	Breast subtype (HR/HER2 status), grade, primary site (quadrant), sex, T/N stage, brain/liver/lung metastasis	-	-	-	Insurance, marital status	American Indian/Alaskan Native, Asian/Pacific Islander, Black, White	-	Risk of bone metastasis	Physicians	Internal, External [69]	-	11
Huang et al. 2020 [69] (Limited availability; accessible through results section in paper)	To develop a predictive model by analyzing SEER data to determine overall survival rates for bone metastases in patients with IDC	Male and female patients with newly diagnosed IDC	SEER database	Multivariate Cox regression	Age, brain/liver metastasis, breast subtype (HR/HER2 status), grade	Chemotherapy, surgery	-	-	Insurance, marital status	Black, Other, White	-	1-, 3-, and 5-year overall survival	Physicians	Internal, External [69]	-	11
Li et al. 2021 [70] (Limited availability; accessible through results section in paper)	To construct nomograms that can predict overall survival of patients with lymph node positive, luminal A breast cancer	Adult (18+) female patients with lymph node positive, luminal A breast cancer	SEER database	Multivariate Cox regression	Age, grade, T stage, number of nodes, bone/brain/liver metastases	Chemotherapy, radiotherapy, surgery	-	-	Marital status	Black, Other, White	-	1-, 3-, and 5-year overall survival	Physicians	Internal [70]	-	11
Li et al. 2021 [70] (Limited availability; accessible through results section in paper)	To construct nomograms that can predict breast cancer-specific survival of patients with lymph node positive, luminal A breast cancer	Adult (18+) female patients with lymph node positive, luminal A breast cancer	SEER database	Multivariate Cox regression	Age, grade, T stage, number of nodes, bone/brain/liver metastases	Chemotherapy, radiotherapy, surgery	-	-	Marital status	Black, Other, White	-	1-, 3-, and 5-year breast cancer-specific survival	Physicians	Internal [70]	-	11
METSSS [71, 146]*	To predict survival after palliative radiotherapy	Male and female cancer patients undergoing palliative radiation therapy during the initial course of treatment	NCDB	Multivariate Cox regression	Age, bone/brain/liver/lung metastases, Charlson-Deyo comorbidity score, radiotherapy site, sex	-	-	-	-	-	-	1- and 5-year overall survival	Physicians	Internal, External [71, 147, 148]	-	29
Nomogram for Locally Advanced Breast Cancer after Immediate Breast Reconstruction [72, 149]	To develop a nomogram that predicts survival outcomes for locally advanced breast cancer patients undergoing immediate breast reduction surgery	Women aged 20–80 years with locally advanced breast cancer stage 3A to 3C undergoing immediate breast reconstruction	SEER database	Multivariate Cox regression (LASSO method)	Age, ER/PR status, grade, HER2 status, T/N stage	Chemotherapy, radiotherapy	-	-	Marital status	-	-	Overall survival	Physicians and patients	Internal [72]	-	15
Nomogram of Cancer-Specific Survival in Male Breast Cancer Patients with Bone Metastases [73, 150]	To develop a nomogram that can accurately predict cancer-specific survival outcomes of male breast cancer patients with bone metastases	Male breast cancer patients with bone metastases	SEER database	Multivariate Cox regression	Age, ER/PR status, T stage	Surgery	-	-	-	-	-	Cancer-specific survival	Physicians and patients	Internal [73]	-	12
Nomogram of Diagnosis of Bone metastases in Male Breast Cancer Patients [73, 151]	To develop a nomogram that can accurately predict risk of bone metastasis in male breast cancer patients	Male breast cancer patients with bone metastases	SEER database	Multivariate Cox regression	Age, T/N stage, tumor size	-	-	-	Marital status	-	-	Risk of bone metastasis	Physicians and patients	Internal [73]	-	12
Nomogram of Overall Survival in Male Breast Cancer Patients with Bone Metastases [73, 152]	To develop a nomogram that can accurately predict overall survival outcomes of male breast cancer patients with bone metastases	Male breast cancer patients with bone metastases	SEER database	Multivariate Cox regression	Age, ER/PR status, T stage	Surgery	-	-	-	-	-	Overall survival	Physicians and patients	Internal [73]	-	12
Nomogram Predicting Survival of Elderly Locally Advanced Breast Cancer [74, 153]	To develop a model that predicts survival outcomes for locally advanced breast cancer in the elderly	Male and female elderly patients (65+) with locally advanced breast cancer (T3N0M0 and Stage III)	SEER database	Multivariate Cox regression (LASSO method)	Age, ER/PR status, grade, T/N stage	Chemotherapy, radiotherapy, surgery	-	-	Marital status	-	-	Overall survival	Physicians	Internal [74]	-	14
PersonalRT27 [82, 154]	To identify parameters that allow patients to be categorized into risk groups and create a locoregional recurrence risk classification tool	Male and female breast cancer patients less than 75 years, cancer stage T1-T2, N0-N1, weak or moderate ER expression, negative, weak, or moderate PR expression, and negative HER2 expression who had undergone surgery and had received systemic adjuvant treatment with hormone therapy, chemotherapy, or locoregional radiotherapy	Public health departments in the Valencian Community (Spain)	Logistic regression analysis	Age, BMI, boost, diabetes, grade, hypertension, Ki 67, lymph node status, lymphovascular invasion, margins, molecular platform result, molecular subtype, pathological anatomy, tumor size	Chemotherapy, radiotherapy	-	Smoking status	-	-	-	Risk of recurrence	Physicians	-	-	11
Resources and Education for Adherence to Cancer Hormonal Therapy (REACH) [80] (Limited availability; accessible through Appendix in paper)	To develop a web-based intervention that used personal values to enhance adjuvant endocrine therapy adherence	Women aged 21 or older with stage 0 to 3 hormone receptor-positive breast cancer survivors with at least moderate difficulty adhering to AET	Pueblo, Boulder, and Colorado Springs clinics of Rocket Mountain Cancer Centers	Independent t-tests, Chi-square tests, and fractional logit models	Side effects of AET	-	-	-	-	-	Values that motivate participants to take care of themselves	1. Monthly adherence to AET 2. Affective attitudes about AET	Patients	-	Yes [80]	-^
Stage 4-Breast Cancer Patients [86, 155]	To create an artificial intelligence model that predicts the five-year survival in patients with stage IV metastatic breast cancer	Patients with stage IV metastatic breast cancer	Cancer registry from the Dongnam Institute of Radiology and Medical Sciences	Artificial intelligence modeling	Age, H-SMA, H2-SMA, hypertension, mellitus, muscle mass, SMA, W-SMA	Chemotherapy, radiotherapy	-	-	-	-	-	5-year survival	Physicians	Internal [86]	-	11
Sun et al. 2020 [16] (Limited availability; accessible through results section in paper)	To establish comprehensive and practical nomograms to accurately predict overall survival for young breast cancer patients	Male and female patients diagnosed with breast cancer before the age of 40 with histologically confirmed ductal or lobular carcinoma	SEER database	Multivariate Cox regression	ER/PR status, grade, HER2 status, location of tumor (quadrant), lymph node ratio, TNM stage	Surgery	-	-	Insurance, marital status	Black, Other/NOS, White	-	3- and 5-year overall survival	Physicians	Internal [16]	-	13
Sun et al. 2020 [16] (Limited availability; accessible through results section in paper)	To establish comprehensive and practical nomograms to accurately predict breast cancer-specific survival for young breast cancer patients	Male and female patients diagnosed with breast cancer before the age of 40 with histologically confirmed ductal or lobular carcinoma	SEER database	Multivariate Cox regression	ER/PR status, grade, HER2 status, location of tumor (quadrant), lymph node ratio, TNM stage	Surgery	-	-	Insurance, marital status	Black, Other/NOS, White	-	3- and 5-year breast cancer-specific survival	Physicians	Internal [16]	-	13
Surrogate Nomogram for OncotypeDX Recurrence Score [83, 156]	To develop a nomogram that can be used to predict the results of the 21-gene recurrence score assay	Patients diagnosed with ER+/HER2− early-stage breast cancer who underwent recurrence score testing	Galway University Hospital, Republic of Ireland	Multivariable logistic regression	Age at diagnosis, ER/PR status, grade, menopausal status, symptomatic status, T stage	-	-	-	-	-	-	Predicted OncotypeDX score	Physicians	Internal [83]	-	14
Wang et al. 2022 [75] (Limited availability; accessible through results section in paper)	To develop nomograms to predict overall survival in patients with breast cancer lung metastases	Male and female adult (18+) patients with breast cancer with positive follow-up and lung metastasis at presentation, breast cancer as the only diagnosed or 1st of 2 of more primary cancer	SEER database	Multivariate Cox regression	Age, grade, laterality, subtype (HR/HER2 status), T stage, bone/brain/liver metastases,	Chemotherapy, surgery	-	-	Marital status	Black, Other, White	-	1-, 2-, and 3-year overall survival	Physicians	Internal [75]	-	11
Wang et al. 2022 [75] (Limited availability; accessible through results section in paper)	To develop nomograms to predict breast cancer-specific survival in patients with breast cancer lung metastases	Male and female adult (18+) patients with breast cancer with positive follow-up and lung metastasis at presentation, breast cancer as the only diagnosed or 1st of 2 of more primary cancer	SEER database	Multivariate Cox regression	Age, grade, laterality, subtype (HR/HER2 status), T stage, bone/brain/liver metastases	Chemotherapy, surgery	-	-	Marital status	Black, Other, White	-	1-, 2-, and 3-year breast cancer-specific survival	Physicians	Internal [75]	-	11
Wu et al. 2022 [76] (Limited availability; accessible through results section in paper)	To predict overall survival in patients with non-metastatic HER2 positive breast cancer	Females with non-metastatic HER2-positive breast cancer	SEER database	Multivariate Cox regression	Age, ER/PR status, site of tumor (quadrant), T/N stage	Chemotherapy, radiation, surgery	-	-	Marital status	Black, Other, White	-	3- and 5-year overall survival	Physicians	Internal, External [76]	-	11
Xu et al. 2022 [77] (Limited availability; accessible through results section in paper)	To establish and validate a new prediction model to predict future triple negative apocrine carcinoma patients	Adult (18+) male and female patients with triple-negative apocrine carcinoma	SEER database	Multivariate Cox regression	Age, first malignancy, stage	Chemotherapy, surgery	-	-	-	-	-	3- and 5-year overall survival	Physicians	Internal [77]	-	11
Young, Empowered, and Strong (YES) [78] (Limited availability; accessible through supplemental information in paper)	To promote the self-monitoring and management of symptoms and concerns in women with young breast cancer	Young women (18–44 years) with breast cancer	30 patients from the Dana-Farber Cancer Institute with newly diagnosed early breast cancer, breast cancer survivors, or metastatic breast cancer	Tailored information based on care interventions and symptom management	Emotional symptoms (anxiety and depression, etc.), physical symptoms (pain and discomfort, vaginal dryness, etc.)	-	-	Adherence to treatment, daily activities, mobility, physical activity, self-care, smoking, and alcohol use	Education, employment, financial status/burden	-	Individual concerns/values	Symptom management	Patients	-	Yes[78]	8
Yin et al. 2022 [79] (Limited availability; accessible through results section in paper)	To establish nomograms for assessing overall survivorship in locally advanced breast cancer patients	Adult (18+) male and female patients with locally advanced breast cancer	SEER database	Multivariate Cox regression (LASSO method)	Age, breast subtype (luminal A or B, HER2, triple-negative), grade, T/N stage	Chemotherapy, radiation, surgery	-	-	Marital status	-	-	1-, 3-, and 5-year overall survival	Physicians	Internal, External [79]	-	11
Yin et al. 2022 [79] (Limited availability; accessible through results section in paper)	To establish nomograms for assessing breast-cancer specific survivorship in locally advanced breast cancer patients	Adult (18+) male and female patients with locally advanced breast cancer	SEER database	Multivariate Cox regression (LASSO method)	Age, breast subtype (luminal A or B, HER2, triple-negative), grade, T/N stage	Chemotherapy, radiation, surgery	-	-	Marital status	-	-	1-, 3-, and 5-year breast cancer-specific survival	Physicians	Internal, External [79]	-	11

*AET* adjuvant endocrine therapy, *AT* aromatase inhibitor, *BCSC* Breast Cancer Surveillance Consortium, *BMI* body mass index, *DCIS* ductal carcinoma in situ, *ER* estrogen receptor, *HER2* human epidermal growth factor receptor 2, *HR* hormone receptor, *H-SMA* height-relative skeletal muscle area, *H2-SMA* height square-relative skeletal muscle area, *IDC* infiltrating ductal carcinoma, *IMPC* invasive micropapillary carcinoma, *Ki 67* Antigen Ki 67, *METABRIC* Molecular Taxonomy of Breast Cancer International Consortium, *M stage* metastasis, *NCDB* National Cancer Database, *N stage* nodal status, *NOS* not otherwise specified, *PR* progesterone receptor, *SEER* Surveillance, Epidemiology, and End Results Program, *SMA* skeletal muscle area, *TRAM* transverse rectus abdominis muscle, *T stage* tumor stage, *W-SMA* weight-relative skeletal muscle area

- = None

* ‘METSSS’[71] was last updated in January 2023

^ Graetz et al. 2018[81] and ‘REACH’[80] were excluded; authors could not accurately assess the full tool

**Table 3:** Key strengths and weaknesses of treatment and survivorship web-based decision-making tools available from 2013 to 2023

Strengths	Weaknesses

• Tools underwent internal and/or external validation • Used plain language at an appropriate reading level • Used event rates to describe outcome probabilities • Adequately described health condition • Tools considering multiple outcomes	• Limited instructions on how to incorporate patient preferences and values in to shared decision making • Missing citations, author credentials, and steps of development • Limited usability, feasibility, and acceptability testing of the tools • Limited validation and usability testing in underserved and underrepresented populations

## Data Availability

Data sharing is not applicable to this article as no datasets were analyzed or generated during the current study. All the studies summarized in this scoping review are listed in the data supplement.
