# Supplementary material for: A scoping review of web-based, interactive, personalized decision-making tools available to support breast cancer treatment and survivorship care
Source: J Cancer Surviv. 2024 Mar 28;19(5):1496–524. doi: 10.1007/s11764-024-01567-6 (PMC11436482; doi:10.1007/s11764-024-01567-6)
Supplement: Supplementary file 1 — Supplementary file1 (DOCX 104 KB) [file 11764_2024_1567_MOESM1_ESM.docx]

**Supplementary Table 1. Preferred Reporting Items for Systematic Reviews and Meta-Analyses extension for Scoping Reviews (PRISMA-ScR) Checklist**

| SECTION | ITEM | PRISMA-ScR CHECKLIST ITEM | REPORTED ON PAGE # |
| --- | --- | --- | --- |
| TITLE | | | |
| Title | 1 | Identify the report as a scoping review. | 1 |
| ABSTRACT | | | |
| Structured summary | 2 | Provide a structured summary that includes (as applicable): background, objectives, eligibility criteria, sources of evidence, charting methods, results, and conclusions that relate to the review questions and objectives. | 2 |
| INTRODUCTION | | | |
| Rationale | 3 | Describe the rationale for the review in the context of what is already known. Explain why the review questions/objectives lend themselves to a scoping review approach. | 3-5 |
| Objectives | 4 | Provide an explicit statement of the questions and objectives being addressed with reference to their key elements (e.g., population or participants, concepts, and context) or other relevant key elements used to conceptualize the review questions and/or objectives. | 5 |
| METHODS | | | |
| Protocol and registration | 5 | Indicate whether a review protocol exists; state if and where it can be accessed (e.g., a Web address); and if available, provide registration information, including the registration number. | 5 |
| Eligibility criteria | 6 | Specify characteristics of the sources of evidence used as eligibility criteria (e.g., years considered, language, and publication status), and provide a rationale. | 6 |
| Information sources* | 7 | Describe all information sources in the search (e.g., databases with dates of coverage and contact with authors to identify additional sources), as well as the date the most recent search was executed. | 6 |
| Search | 8 | Present the full electronic search strategy for at least 1 database, including any limits used, such that it could be repeated. | S4-7 |
| Selection of sources of evidence† | 9 | State the process for selecting sources of evidence (i.e., screening and eligibility) included in the scoping review. | 6-8 |
| Data charting process‡ | 10 | Describe the methods of charting data from the included sources of evidence (e.g., calibrated forms or forms that have been tested by the team before their use, and whether data charting was done independently or in duplicate) and any processes for obtaining and confirming data from investigators. | 6-8 |
| Data items | 11 | List and define all variables for which data were sought and any assumptions and simplifications made. | 6-8 |
| Critical appraisal of individual sources of evidence§ | 12 | If done, provide a rationale for conducting a critical appraisal of included sources of evidence; describe the methods used and how this information was used in any data synthesis (if appropriate). | 8 |
| Synthesis of results | 13 | Describe the methods of handling and summarizing the data that were charted. | 6-8 |
| RESULTS | | | |
| Selection of sources of evidence | 14 | Give numbers of sources of evidence screened, assessed for eligibility, and included in the review, with reasons for exclusions at each stage, ideally using a flow diagram. | 8 |
| Characteristics of sources of evidence | 15 | For each source of evidence, present characteristics for which data were charted and provide the citations. | 8-13 |
| Critical appraisal within sources of evidence | 16 | If done, present data on critical appraisal of included sources of evidence (see item 12). | 13-14 |
| Results of individual sources of evidence | 17 | For each included source of evidence, present the relevant data that were charted that relate to the review questions and objectives. | 33-40 |
| Synthesis of results | 18 | Summarize and/or present the charting results as they relate to the review questions and objectives. | 14 |
| DISCUSSION | | | |
| Summary of evidence | 19 | Summarize the main results (including an overview of concepts, themes, and types of evidence available), link to the review questions and objectives, and consider the relevance to key groups. | 14-19 |
| Limitations | 20 | Discuss the limitations of the scoping review process. | 19 |
| Conclusions | 21 | Provide a general interpretation of the results with respect to the review questions and objectives, as well as potential implications and/or next steps. | 20 |
| FUNDING | | | |
| Funding | 22 | Describe sources of funding for the included sources of evidence, as well as sources of funding for the scoping review. Describe the role of the funders of the scoping review. | 21 |

JBI = Joanna Briggs Institute; PRISMA-ScR = Preferred Reporting Items for Systematic reviews and Meta-Analyses extension for Scoping Reviews.

* Where *sources of evidence* (see second footnote) are compiled from, such as bibliographic databases, social media platforms, and Web sites.

† A more inclusive/heterogeneous term used to account for the different types of evidence or data sources (e.g., quantitative and/or qualitative research, expert opinion, and policy documents) that may be eligible in a scoping review as opposed to only studies. This is not to be confused with *information sources* (see first footnote).

‡ The frameworks by Arksey and O’Malley^31^ and Levac and colleagues^32^ and the JBI guidance^33^ refer to the process of data extraction in a scoping review as data charting*.*

§ The process of systematically examining research evidence to assess its validity, results, and relevance before using it to inform a decision. This term is used for items 12 and 19 instead of "risk of bias" (which is more applicable to systematic reviews of interventions) to include and acknowledge the various sources of evidence that may be used in a scoping review (e.g., quantitative and/or qualitative research, expert opinion, and policy document).

*From:* Tricco AC, Lillie E, Zarin W, O'Brien KK, Colquhoun H, Levac D, et al. PRISMA Extension for Scoping Reviews (PRISMAScR): Checklist and Explanation. Ann Intern Med. 2018;169:467–473.

**Supplementary Table 2. Database Search Strategies**

Database: PubMed/MEDLINE
Platform: National Library of Medicine
Date Searched: 5/12/2023

Date Limits: 1/1/2013 – 5/12/2023

Limits: English & Human

|  | Concept: | Search Strategy: |
| --- | --- | --- |
| #1 | Breast Cancer | "Breast Neoplasms"[Mesh] OR "breast neoplasm*”[Title/Abstract] OR “breast cancer"[Title/Abstract:~4] OR “breast cancers"[Title/Abstract:~4] OR "breast carcinoma*"[Title/Abstract] OR "breast tumor*”[Title/Abstract] OR “breast tumour*"[Title/Abstract] OR "breast sarcoma*”[Title/Abstract] |
| #2 | Risk prediction models/ personalized clinical tools | "personalized*"[Title/Abstract] OR "decision mak*"[Title/Abstract] OR "individualized*"[Title/Abstract] OR "Decision Support Techniques"[Mesh] OR "decision aid*"[Title/Abstract] OR "calculator*"[Title/Abstract] OR (("decision*"[Title/Abstract] OR "predict*"[Title/Abstract] OR "prognos*"[Title/Abstract] OR "risk*"[Title/Abstract] OR "clinical*"[Title/Abstract] OR "interactive"[Title/Abstract]) AND ("tool*"[Title/Abstract] OR "model*"[Title/Abstract] OR "technolog*"[Title/Abstract] OR "technique*"[Title/Abstract] OR "instrument*"[Title/Abstract] OR "stratif*"[Title/Abstract] OR "algorithm*"[Title/Abstract] OR "nomogram*"[Title/Abstract] OR "scor*"[Title/Abstract])) |
| #3 | Online/web-based calculator | "Internet"[Mesh] OR "internet*"[Title/Abstract] OR "web"[Title/Abstract] OR "online"[Title/Abstract] OR "Mobile Applications"[Mesh] OR "app"[Title/Abstract] OR "apps"[Title/Abstract] OR "mobile*"[Title/Abstract] OR "Telemedicine"[Mesh] OR "telemedicine"[Title/Abstract] OR "telehealth"[Title/Abstract] OR "tele-health"[Title/Abstract] OR "mhealth"[Title/Abstract] OR "m-health"[Title/Abstract] OR "ehealth"[Title/Abstract] OR “e-health"[Title/Abstract] OR "Microcomputers"[Mesh] OR "microcomputer*"[Title/Abstract] OR "personal computer*"[Title/Abstract] OR "handheld computer*"[Title/Abstract] OR "smartphone*"[Title/Abstract] OR "smart-phone*"[Title/Abstract] OR "cellphone*"[Title/Abstract] OR "cell-phone*"[Title/Abstract] OR "iPhone*"[Title/Abstract] OR "iPad*"[Title/Abstract] OR "tablet*"[Title/Abstract] OR "android*"[Title/Abstract] |
| #4 | Limits & Filters | ((#1 AND #2 AND #3) NOT ("Animals"[Mesh] NOT ("Animals"[Mesh] AND "Humans"[Mesh]))) NOT (editorial[Publication Type] OR comment[Publication Type] OR "commentary*"[Title/Abstract] OR news[Publication Type] OR letter[Publication Type] OR retracted publication[Publication Type] OR retraction of publication[Publication Type] OR "retraction of publication*"[Title/Abstract] OR "retraction notice"[Title] OR "retracted publication"[Title] OR "Congress"[Publication Type] OR "Consensus Development Conference"[Publication Type] OR "conference abstract*"[Title/Abstract] OR "conference proceeding*"[Title/Abstract] OR "conference paper*"[Title/Abstract] OR "conference review*"[Title/Abstract] OR "symposium*"[Title/Abstract] OR "Case Reports" [Publication Type] OR "case report*"[Title] OR "Review" [Publication Type] OR "Systematic Review" [Publication Type] OR "Systematic Reviews as Topic"[Mesh] OR "systematic review"[Title/Abstract] OR "Meta-Analysis" [Publication Type] OR "Meta-Analysis as Topic"[Mesh] OR "meta-analysis "[Title] OR "meta-analyses "[Title] OR "protocol"[Title] OR "protocols"[Title] OR "Practice Guideline"[Publication Type] OR "guideline*"[Title]) Filters: English, from 2013/1/1 - 2023/5/12 |

Database: Embase
Platform: Elsevier
Date Searched: 5/12/2023

Date Limits: 1/1/2013 – 5/12/2023

Limits: English & Human

|  | Concept: | Search Strategy: |
| --- | --- | --- |
| #1 | Breast Cancer | 'breast cancer'/exp OR 'breast neoplasm*':ab,ti OR ((breast NEAR/4 cancer*):ab,ti) OR 'breast carcinoma*':ab,ti OR 'breast tumor*':ab,ti OR 'breast tumour*':ab,ti OR 'breast sarcoma*':ab,ti |
| #2 | Risk prediction models/ personalized clinical tools | 'decision support system'/exp OR 'personalized*':ab,ti OR 'decision mak*':ab,ti OR 'individualized*':ab,ti OR 'decision aid*':ab,ti OR 'calculator*':ab,ti OR ((('decision*' OR 'predict*' OR 'prognos*' OR 'risk*' OR 'clinical*' OR 'interactive') NEAR/4 ('tool*' OR 'model*' OR 'technolog*' OR 'technique*' OR 'instrument*' OR 'stratif*' OR 'algorithm*' OR 'nomogram*' OR 'scor*')):ab,ti) |
| #3 | Online/web-based calculator | 'internet'/exp OR 'mobile application'/exp OR 'telemedicine'/exp OR 'microcomputer'/exp OR 'internet*':ab,ti OR 'web':ab,ti OR 'online':ab,ti OR 'app':ab,ti OR 'apps':ab,ti OR 'mobile*':ab,ti OR 'telemedicine':ab,ti OR 'telehealth':ab,ti OR 'tele-health':ab,ti OR 'mhealth':ab,ti OR 'm-health':ab,ti OR 'ehealth':ab,ti OR 'e-health':ab,ti OR 'microcomputer*':ab,ti OR 'personal computer*':ab,ti OR 'handheld computer*':ab,ti OR 'smartphone*':ab,ti OR 'smart-phone*':ab,ti OR 'cellphone*':ab,ti OR 'cell-phone*':ab,ti OR 'iphone*':ab,ti OR 'ipad*':ab,ti OR 'tablet*':ab,ti OR 'android*':ab,ti |
| #4 | Limits & Filters | #1 AND #2 AND #3 AND [english]/lim AND [2013-2023]/py NOT ([animals]/lim NOT ([animals]/lim AND [humans]/lim)) NOT ([conference abstract]/lim OR [conference paper]/lim OR [conference review]/lim OR 'conference abstract*':ab,ti OR 'conference proceeding*':ab,ti OR [editorial]/lim OR 'retraction notice'/exp OR 'retraction of publication':ab,ti OR 'retraction notice':ti OR 'retracted publication':ab,ti OR [letter]/lim OR [note]/lim OR 'case report'/exp OR 'case report':ti OR 'practice guideline'/exp OR guideline*:ti OR [review]/lim OR [systematic review]/lim OR 'systematic review':ti OR [meta analysis]/lim OR 'meta analysis':ti OR 'meta-analyses':ti) |

Database: Cochrane CENTRAL
Platform: Wiley & Sons
Date Searched: 5/12/2023

Date Limits: 1/1/2013 – 5/12/2023

Limits: English & Human

|  | Concept: | Search Strategy: |
| --- | --- | --- |
| #1 | Breast Cancer | ([mh "Breast Neoplasms"] OR "breast neoplasm*” OR breast NEAR/4 cancer* OR "breast carcinoma*" OR "breast tumor*" OR "breast tumour*" OR "breast sarcoma*"):ti,ab,kw |
| #2 | Risk prediction models/ personalized clinical tools | ("personalized*" OR "decision mak*" OR "individualized*" OR [mh "Decision Support Techniques"] OR "decision aid*" OR "calculator*" OR (("decision*" OR "predict*" OR "prognos*" OR "risk*" OR "clinical*" OR "interactive") NEAR/4 ("tool*" OR "model*" OR "technolog*" OR "technique*" OR "instrument*" OR "stratif*" OR "algorithm*" OR "nomogram*" OR "scor*"))):ti,ab,kw" |
| #3 | Online/web-based calculator | ([mh "Internet"] OR "internet*" OR "web" OR "online" OR [mh "Mobile Applications"] OR "app" OR "apps" OR "mobile*" OR [mh "Telemedicine"] OR "telemedicine" OR "telehealth" OR "tele-health" OR "mhealth" OR "m-health" OR "ehealth" OR "e-health" OR [mh "Microcomputers"] OR "microcomputer*" OR "personal computer*" OR "handheld computer*" OR "smartphone*" OR "smart-phone*" OR "cellphone*" OR "cell-phone*" OR "iPhone*" OR "iPad*" OR "tablet*" OR "android*"):ti,ab,kw |
| #4 | Limits & Filters | #1 AND #2 AND #3" with Publication Year from 2013 to 2023, in Trials (Word variations have been searched) |

Database: Web of Science (Core Collection)
Platform: Clarivate Analytics
Date Searched: 5/12/2023

Date Limits: 1/1/2013 – 5/12/2023

Limits: English & Human

|  | Concept: | Search Strategy: |
| --- | --- | --- |
| #1 | Breast Cancer | TS=("breast neoplasm*” OR breast NEAR/4 cancer* OR "breast carcinoma*" OR "breast tumor*” OR "breast tumour*" OR "breast sarcoma*") |
| #2 | Risk prediction models/ personalized clinical tools | TS=("personalized*" OR "decision mak*" OR "individualized*" OR "decision aid*" OR "calculator*" OR (("decision*" OR "predict*" OR "prognos*" OR "risk*" OR "clinical*" OR "interactive") NEAR/4 ("tool*" OR "model*" OR "technolog*" OR "technique*" OR "instrument*" OR "stratif*" OR "algorithm*" OR "nomogram*" OR "scor*"))) |
| #3 | Online/web-based calculator | TS=("internet*" OR "web" OR "online" OR "app" OR "apps" OR "mobile*" OR "telemedicine" OR "telehealth" OR "tele-health" OR "mhealth" OR "m-health" OR "ehealth" OR "e-health" OR "microcomputer*" OR "personal computer*" OR "handheld computer*" OR "smartphone*" OR "smart-phone*" OR "cellphone*" OR "cell-phone*" OR "iPhone*" OR "iPad*" OR "tablet*" OR "android*") |
| #4 | Limits & Filters | #3 AND #2 AND #1 and Web of Science Core Collection (Database) and Editorial Material OR Letter OR Meeting OR Meeting Abstract OR Item Withdrawal OR News Item OR Note OR Proceedings Paper OR Retracted Publication OR Retraction OR Review OR Withdrawn Publication (Document Type) Meeting or Review Article (Exclude – DocumentTypes) and Animals or Mice or Mice Nude (Exclude – MeSH Headings) and English (Languages) \| Timespan: 2013-01-01 to 2023-05-12 (Publication Date) |

Database: Scopus
Platform: Elsevier
Date Searched: 5/12/2023

Date Limits: 1/1/2013 – 5/12/2023

Limits: English & Human

|  | Concept: | Search Strategy: |
| --- | --- | --- |
| #1 | Breast Cancer | ( TITLE-ABS-KEY ( "breast neoplasm*" OR breast W/4 cancer* OR "breast carcinoma*" OR "breast tumor*" OR "breast tumour*" OR "breast sarcoma*" ) |
| #2 | Risk prediction models/ personalized clinical tools | TITLE-ABS-KEY ("personalized*" OR "decision mak*" OR "individualized*" OR "decision aid*" OR "calculator*" OR "decision*" OR "predict*"OR "prognos*"  OR "risk*" OR "clinical*" OR "interactive" W/4 "tool*" OR "model*" OR "technolog*" OR "technique*" OR "instrument*" OR "stratif*" OR "algorithm*" OR "nomogram*" OR "scor*") |
| #3 | Online/web-based calculator | TITLE-ABS-KEY ("internet*" OR "web" OR "online" OR "app" OR "apps" OR "mobile*" OR "telemedicine" OR "telehealth" OR "tele-health" OR "mhealth" OR "m-health" OR "ehealth" OR "e-health" OR "microcomputer*" OR "personal computer*" OR "handheld computer*" OR "smartphone*" OR "smart-phone*" OR "cellphone*" OR "cell-phone*" OR "iphone*" OR "ipad*" OR "tablet*" OR "android*" ) |
| #4 | Limits & Filters | AND LANGUAGE (english) ) AND PUBYEAR > 2012 AND PUBYEAR < 2024 AND (EXCLUDE (DOCTYPE, "re") OR EXCLUDE ( DOCTYPE , "cp" ) OR EXCLUDE ( DOCTYPE , "cr" ) OR EXCLUDE ( DOCTYPE , "ch" ) OR EXCLUDE ( DOCTYPE , "ed" ) OR EXCLUDE ( DOCTYPE , "le" ) OR EXCLUDE ( DOCTYPE , "bk" ) OR EXCLUDE ( DOCTYPE , "no" ) |

Database: PsycINFO
Vendor: American Psychological Association
Date Searched: 5/12/2023

Date Limits: 1/1/2013 – 5/12/2023

Limits: English & Human

|  | Concept: | Search Strategy: |
| --- | --- | --- |
| #1 | Breast Cancer AND Risk prediction models/ personalized clinical tools  AND  Online/web-based calculator  Limits & Filters | (IndexTermsFilt:("Breast Neoplasms")) OR (Title:("breast neoplasm*") OR Title:(breast NEAR/4 cancer*) OR Title:("breast carcinoma*") OR Title:("breast tumor*") OR Title:("breast tumour*") OR Title:("breast sarcoma*")) OR Abstract:("breast neoplasm*" OR breast NEAR/4 cancer* OR "breast carcinoma*" OR "breast tumor*" OR "breast tumo)) AND ((PublicationYear:[2013 TO 2023]) AND PublicationTypeFilt:"Peer Reviewed Journal")) AND (((IndexTermsFilt: ("Decision Support Systems")) OR (title: ("personalized*") OR title: ("decision mak*") OR title: ("individualized*") OR title: ("decision aid*") OR title: ("calculator*") OR ((title: ("decision*") OR title: ("predict*") OR title: ("prognos*") OR title: ("risk*") OR title: ("clinical*") OR title: ("interactive")) NEAR/4 (title: ("tool*") OR title: ("model*") OR title: ("technolog*") OR title: ("technique*") OR title: ("instrument*") OR title: ("stratif*") OR title: ("algorithm*") OR title: ("nomogram*") OR title: ("scor*")))) OR (abstract: ("personalized*") OR abstract: ("decision mak*") OR abstract: ("individualized*") OR abstract: ("decision aid*") OR abstract: ("calculator*") OR ((abstract: ("decision*") OR abstract: ("predict*") OR abstract: ("prognos*") OR abstract: ("risk*") OR abstract: ("clinical*") OR abstract: ("interactive")) NEAR/4 (abstract: ("tool*") OR abstract: ("model*") OR abstract: ("technolog*") OR abstract: ("technique*") OR abstract: ("instrument*") OR abstract: ("stratif*") OR abstract: ("algorithm*") OR abstract: ("nomogram*") OR abstract: ("scor*"))))) AND ((Year: [2013 TO 2023]) AND PublicationTypeFilt: "Peer Reviewed Journal")) ("Internet") OR IndexTermsFilt: ("Mobile Applications") OR IndexTermsFilt: ("Telemedicine") OR IndexTermsFilt: ("Microcomputers")) OR (title: ("internet*") OR title: ("web") OR title: ("online") OR title: ("app") OR title: ("apps") OR title: ("mobile*") OR title: ("telemedicine") OR title: ("telehealth") OR title: ("tele-health") OR title: ("mhealth") OR title: ("m-health") OR title: ("ehealth") OR title: ("e-health") OR title: ("microcomputer*") OR title: ("personal computer*") OR title: ("handheld computer*") OR title: ("smartphone*") OR title: ("smart-phone*") OR title: ("cellphone*") OR title: ("cell-phone*") OR title: ("iPhone*") OR title: ("iPad*") OR title: ("tablet*") OR title: ("android*")) OR (abstract: ("internet*") OR abstract: ("web") OR abstract: ("online") OR abstract: ("app") OR abstract: ("apps") OR abstract: ("mobile*") OR abstract: ("telemedicine") OR abstract: ("telehealth") OR abstract: ("tele-health") OR abstract: ("mhealth") OR abstract: ("m-health") OR abstract: ("ehealth") OR abstract: ("e-health") OR abstract: ("microcomputer*") OR abstract: ("personal computer*") OR abstract: ("handheld computer*") OR abstract: ("smartphone*") OR abstract: ("smart-phone*") OR abstract: ("cellphone*") OR abstract: ("cell-phone*") OR abstract: ("iPhone*") OR abstract: ("iPad*") OR abstract: ("tablet*") OR abstract: ("android*"))) AND ((Year: [2013 TO 2023]) AND PublicationTypeFilt: "Peer Reviewed Journal")) |

**Supplementary Table 3. Inclusion and Exclusion Criteria for Scoping Review**

| Criteria | Inclusion | Exclusion |
| --- | --- | --- |
| Population | - Adults with breast cancer (18+) - Individuals living in the U.S. | - Non-human subjects |
| Concept | - Breast cancer treatment or survivorship - Online web-based risk prediction models, interactive and personalized clinical tools | - Not related to breast cancer - Breast cancer prevention or screening |
| Context | - U.S. (Including online studies) - 2013-2023 | - Studies in countries outside of the U.S. using a non-U.S. population |
| Type of Evidence | - Primary empirical research studies (e.g., randomized controlled trials, cohort studies, and cross-sectional studies) - Full-text articles - Articles written in English | - Reviews (e.g., meta-analysis, systematic reviews) - Editorials (e.g., perspectives) - Abstracts, Conference proceedings or posters - Articles for which we cannot obtain the full text. - Articles not in English - Dissertations - Research protocols - Case reports - Patents |

**Supplemental Table 4. International Patient Decision Aids Standards Instrument (IPDASi) Checklist**

| **Item** | **IPDASi dimension** | **Item description** |
| --- | --- | --- |
| 1 | Information about options | Does the patient decision aid describe the health condition? |
| 2 |  | Does the patient decision aid list the options? |
| 3 |  | Does the patient decision aid list the option of doing nothing? |
| 4 |  | Does the patient decision aid describe the natural course without options? |
| 5 |  | Does the patient decision aid describe procedures? |
| 6 |  | Does the patient decision aid describe positive features (benefits)? |
| 7 |  | Does the patient decision aid describe negative features of options (harms/side effects/disadvantages)? |
| 8 |  | Does the patient decision aid include chances of positive/negative outcomes? |
| 9 |  | Does the patient decision aid describe what test is designed to measure? |
| 10 |  | Does the patient decision aid include chances of true positive, true negative, false positive, false negative test results? |
| 11 |  | Does the patient decision aid describe possible next steps based on test result? |
| 12 |  | Does the patient decision aid include chances the disease is found with/without screening? |
| 13 |  | Does the patient decision aid describe detection/treatment that would never have caused problems if one was not screened? |
| 14 | Outcome probabilities | Does the patient decision aid use event rates specifying the population and time period? |
| 15 |  | Does the patient decision aid compare outcome probabilities using the same denominator, time period, scale? |
| 16 |  | Does the patient decision aid describe uncertainty around probabilities? |
| 17 |  | Does the patient decision aid use visual diagrams? |
| 18 |  | Does the patient decision aid use multiple methods to view probabilities (words, numbers, diagrams)? |
| 19 |  | Does the patient decision aid allow the patient to select a way of viewing probabilities (words, numbers, diagrams)? |
| 20 |  | Does the patient decision aid allow patient to view probabilities based on their own situation (e.g., age)? |
| 21 |  | Does the patient decision aid place probabilities in context of other events? |
| 22 |  | Does the patient decision aid use both positive and negative frames (e.g., showing both survival and death rates)? |
| 23 | Clarifying values | Does the patient decision aid describe the procedures and outcomes to help patients imagine what it is like to experience their physical, emotional, social effects? |
| 24 |  | Does the patient decision aid ask patients to consider which positive and negative features matter most? |
| 25 |  | Does the patient decision aid suggest ways for patients to share what matters most with others? |
| 26 | Decision guidance | Does the patient decision aid provide steps to make a decision? |
| 27 |  | Does the patient decision aid suggest ways to talk about the decision with a health professional? |
| 28 |  | Does the patient decision aid include tools (worksheet, question list) to discuss options with others? |
| 29 | Presenting information | Does the patient decision aid compare positive/negative features of outcomes? |
| 30 |  | Does the patient decision aid show negative/positive features with equal detail (font, order, display of statistics)? |
| 31 | Development process | Does the patient decision aid include developers’ credentials/qualifications? |
| 32 |  | Does the patient decision aid find out what users (patients, practitioners) need to discuss options? |
| 33 |  | Does the patient decision aid have peer review by patient/professional experts not involved in development and field testing? |
| 34 |  | Does the patient decision aid have field testing with users (patients facing the decision, practitioners presenting options)? |
| 35 |  | Does the patient decision aid have field testing that shows the patient decision aid is acceptable? |
| 36 |  | Does the patient decision aid have field testing that shows the patient decision aid is balanced for undecided patients? |
| 37 |  | Does the patient decision aid have field testing that shows the patient decision aid is understood by those with limited reading skills? |
| 38 | Using evidence | Does the patient decision aid provide references to evidence used? |
| 39 |  | Does the patient decision aid report steps to find, appraise, summarize evidence? |
| 40 |  | Does the patient decision aid report date of last update? |
| 41 |  | Does the patient decision aid report how often patient decision aid is updated? |
| 42 |  | Does the patient decision aid describe quality of scientific evidence (including lack of evidence)? |
| 43 |  | Does the patient decision aid use evidence from studies of patients similar to those of target audience? |
| 44 | Disclosure and transparency | Does the patient decision aid report source of funding to develop and distribute the patient decision aid? |
| 45 |  | Does the patient decision aid report whether authors or their affiliations stand to gain or lose by choices patients make after using the patient decision aid? |
| 46 | Plain language | Is the patient decision aid written at a level that can be understood by the majority of patients in the target group? |
| 47 |  | Is the patient decision aid written at a grade 8 equivalent level or less according to readability score (SMOG or FRY)? |
| 48 |  | Does the patient decision aid provide ways to help patients understand information other than reading (audio, video, in-person discussion)? |
| 49 | Internet-based | Does the patient decision aid provide a step-by-step way to move through the web pages? |
| 51 |  | Does the patient decision aid provide feedback on personal health information that is entered into the patient decision aid? |
| 52 |  | Does the patient decision aid provide security for personal health information entered into the decision aid? |
| 53 |  | Does the patient decision aid make it easy for patients to return to the decision aid after linking to other web pages? |
|  |  | Does the patient decision aid permit printing as a single document? |
| 54 | Story usage | Does the patient decision aid use stories that represent a range of positive and negative experiences? |
| 55 |  | Does the patient decision aid report if there was a financial or other reason why patients decided to share their story? |
| 56 |  | Does the patient decision aid state in an accessible document that the patient gave informed consent to use their stories? |
| 57 | Decision processes | Does the patient decision aid recognize a decision needs to be made? |
| 58 |  | Does the patient decision aid know options and their features? |
| 59 |  | Does the patient decision aid understand that values affect decisions? |
| 60 |  | Does the patient decision aid help patients be clear about option features that matter most? |
| 61 |  | Does the patient decision aid help patients discuss values with their practitioner? |
| 62 |  | Does the patient decision aid help patients become involved in preferred ways? |
| 63 | Decision quality | Does the patient decision aid improve the match between the chosen option and the features that matter most to the informed patient? |

**Supplemental Table 5. Results of Validation, Usability, Feasibility, and Acceptability**

| **Tool** | **Race/Ethnicity** | | | | | | **Education** | | | **Income Level** | | | | | **Marital Status** | | **Insurance Status** | |
| --- | --- | --- | --- | --- | --- | --- | --- | --- | --- | --- | --- | --- | --- | --- | --- | --- | --- | --- |
|  | **American Indian/Alaska Native** | **Asian/Pacific Islander** | **Black** | **Hispanic** | **Other*** | **White** | **High School or Less** | **Some College** | **College or Higher** | **0-25^th^ Percentile** | **26-50^th^ Percentile** | **51-75^th^ Percentile** | **76-100^th^ Percentile** | **Missing/Other** | **Married** | **Unmarried** | **Insured** | **Uninsured** |
| **Validation Testing** | | | | | | | | | | | | | | | | | | |
| BreastCHOICE [15, 122] | - | - | - | - | - | - | - | - | - | 24.3% | 24.8% | 24.1% | 23.1% | 3.7% | - | - | - | - |
| BTxChoice [14] (Internal) | - | - | 7.2% | - | Other: 8.3%; Unknown: 0.6% | 83.9% | - | - | - | - | - | - | - | - | - | - | - | - |
| BTxChoice [14] (External) | 2.0% | 11.0% | 7.0% | - | Pacific Islander: <1.0%; Other: 7.0% | 73.0% | - | - | - | - | - | - | - | - | - | - | - | - |
| After Cancer Education and Support Operations [55, 131] | - | - | - | - | 7.0% | 93.0% | 13.0% | - | Associates: 20%, Bachelors: 47%, Masters: 20% | - | - | - | - | - | - | - | - | - |
| Application of Machine Learning Methods to Predict Bone Metastases in Breast Infiltrating Ductal Carcinoma Patients [56, 132] | 0% | 100% | 0% | 0% | 0% | 0% | - | - | - | - | - | - | - | - | 78.0% | 22.0% | - | - |
| Breast Cancer Lung Metastasis Cancer-Specific Survival Nomogram [57, 133] | - | - | 20.0% | - | 7.7% | 72.3% | - | - | - | - | - | - | - | - | 41.4% | 58.6% | 93.7% | 6.3% |
| Breast Cancer Lung Metastasis Overall Survival Nomogram [57, 134] | - | - | 20.0% | - | 7.7% | 72.3% | - | - | - | - | - | - | - | - | 41.4% | 58.6% | 93.7% | 6.3% |
| Breast Cancer Surgery Risk Calculator [59, 137] | 0.4% | 5.6% | 10.2% | 6.3% | Unknown Race: 16.5%  Unknown ethnicity: 15.0% | 67.3% | **-** | **-** | **-** | **-** | **-** | **-** | **-** | **-** | **-** | **-** | - | - |
| Conditional Survival Nomogram [62] | - | - | 19.8% | - | 8.5% | 71.7% | - | - | - | - | - | - | - | - | 57.7% | 42.3% | - | - |
| Dai et. al 2018 [63] | 0.6% | 6.8% | 20.2% | - | - | 72.4% | - | - | - | - | - | - | - | - | 60.1% | 39.9% | - | - |
| Dynamic Nomogram [64, 141] | - | - | 14.4% | - | 6.3% | 79.3% | - | - | - | - | - | - | - | - | 42.6% | 57.4% | - | - |
| Dynamic Nomogram [65, 142] | - | - | 6.6% | - | 6.6% | 86.8% | - | - | - | - | - | - | - | - | 85.6% | 14.4% |  |  |
| Equation [67, 145] | - | - | 11.1% | - | 12.1% | 76.8% | - | - | - | - | - | - | - | - | - | - | - | - |
| Huang et. al 2022 [68] | - | External: 100% | Internal: 15.1% | - | Internal: 13.1% | Internal: 71.8% | - | - | - | - | - | - | - | - | Married: 59.6% | Single: 29.2  Separated/  divorced/  widowed: 7.0%  NOS: 4.2% | - | - |
| Huang et. al 2020 [69] (CSS) | - | - | 12.5% | - | 9.0% | 78.5% | - | - | - | - | - | - | - | - | 52.1% | 47.9% | 94.9% | 5.1% |
| Huang et. al 2020 [69] (OS) | - | - | 12.5% | - | 9.0% | 78.5% | - | - | - | - | - | - | - | - | 52.1% | 47.9% | 94.9% | 5.1% |
| Li et. al 2021 [70] (CSS) | - | - | 10.9% | - | 9.5% | 79.6% | - | - | - | - | - | - | - | - | 59.0% | 41.0% | - | - |
| Li et. al 2021 [70] (OS) | - | - | 10.9% | - | 9.5% | 79.6% | - | - | - | - | - | - | - | - | 59.0% | 41.0% | - | - |
| Nomogram of Cancer-Specific Survival in Male Breast Cancer Patients with Bone Metastases [73, 150] | - | - | 14.6% | - | 6.0% | 79.4% | - | - | - | - | - | - | - | - | 69.3% | 30.7% | - | - |
| Nomogram of Diagnosis of Bone Metastases in Male Breast Cancer Patients [73, 151] | - | - | 14.6% | - | 6.0% | 79.4% | - | - | - | - | - | - | - | - | 69.3% | 30.7% | - | - |
| Nomogram of Overall Survival in Male Breast Cancer Patients with Bone Metastases [73, 152] | - | - | 14.6% | - | 6.0% | 79.4% | - | - | - | - | - | - | - | - | 69.3% | 30.7% | - | - |
| Nomogram Predicting Survival of Elderly Locally Advanced Breast Cancer [74, 153] | - | - | - | - | - | - | - | - | - | - | - | - | - | - | 41.6% | 58.4% | - | - |
| Sun et. al 2020 [16] (CSS) | - | - | - | 15.4% | 13.7% | 70.9% | - | - | - | - | - | - | - | - | 57.3% | Single: 30.4%  Separated/  divorced/  widowed: 6.9%  NOS: 5.4% | 94.7% | 5.3% |
| Sun et. al 2020 [16] (OS) | - | - | - | 15.4% | 13.7% | 70.9% | - | - | - | - | - | - | - | - | 57.3% | Single: 30.4%  Separated/  divorced/  widowed: 6.9%  NOS: 5.4% | 94.7% | 5.3% |
| Wang et. al 2022 [75] (CSS) | - | - | 19.8% | - | 9.3% | 70.9% | - | - | - | - | - | - | - | - | 56.8% | 43.2% | - | - |
| Wang et. al 2022 [75] (OS) | - | - | 19.8% | - | 9.3% | 70.9% | - | - | - | - | - | - | - | - | 56.8% | 43.2% | - | - |
| Wu et. al 2022 [76] | - | - | 12.3% | - | 11.7% | 76.0% | - | - | - | - | - | - | - | - | 59.8% | 40.2% | - | - |
| Xu et. al 2022 [77] | - | - | 10.7% | - | Other: 14.3%  NOS: 1.8% | 73.2% | - | - | - | - | - | - | - | - | - | - | - | - |
| Yin et. al 2022 [79] (CSS) (Population 1) | - | 100% | - | - | - | - | - | - | - | - | - | - | - | - | 50.5% | Single: 23.0%  Separated/  divorced/ widowed: 26.5% | - | - |
| Yin et. al 2022 [79] (CSS) (Population 2) | - | - | 16.0% | - | 10.1% | 73.79% | - | - | - | - | - | - | - | - | 53.1% | Single: 20.4%  Separated/  divorced/  widowed: 26.5% | - | - |
| Yin et. al 2022 [79] (OS) (Population 1) | - | 100% | - | - | - | - | - | - | - | - | - | - | - | - | 50.5% | Single: 23.0%  Separated/  divorced/ widowed: 26.5% | - | - |
| Yin et. al 2022 [79] (OS) (Population 2) | - | - | 16.0% | - | 10.1% | 73.79% | - | - | - | - | - | - | - | - | 53.1% | Single: 20.4%  Separated/  divorced/  widowed: 26.5% | - | - |
| **Usability/Acceptability/Feasibility** | | | | | | | | | | | | | | | | | | |
| BRECONDA [17, 123] | - | - | - | - | - | - | 69.0% | - | - | - | - | - | - | - | 86.0% | 14.0% | - | - |
| BRECONDA [53, 123] | - | - | - | - | - | - | 21.7% | - | Vocational: 25.0%  University: 53.3% | - | - | - | - | - | Living with a partner: 80.0% | Not living with a partner: 20.0% | - | - |
| BRECONDA [54, 123] | - | - | - | - | - | - | 23.8% | - | Vocational: 19.4%  Bachelors: 35.6%  Graduate: 21.1% | - | - | - | - | < $50K: 28.6%  $50K-$90K: 28.1%  >$90K: 43.2% | 71.1% | Single: 9.5%  Separated/  divorced/  widowed: 19.4% | - | - |
| Which treatment for DCIS is right for you? [46] | - | - | 31.8% | - | - | 68.2% | - | - | - | -- | - | - | - | - | - | - | - | - |
| After Cancer Education and Support Operations [55] (Population 1) | - | - | - | - | 7.0% | 93.0% | - | 13.0% | Associates: 20.0%, Bachelors: 47.0%, Masters: 20.0% | - | - | - | - | - | - | - | - | - |
| After Cancer Education and Support Operations [55] (Population 2) | - | - | - | 8.0% | 12.0% | 80.0% | - | - | Associates/Technical: 14.0%  Bachelors: 57.0%  Masters: 23.0%  Doctoral: 6.0% | - | - | - | - | - | 94.0% | Divorced or separated: 6.0% | - | - |
| Graetz et. al 2018 [81] | - | - | 22.7% | 2.3% | - | 75.0% | 5.0% | 55.0% | 40.0% | - | - | - | - | Less than 150% FPL: 36.4%  150-400% FPL: 27.3%  400% FPL or more: 36.4% | 73.8% | Single: 14.3%  Divorced: 4.8%  Widowed: 7.1% | - | - |
| REACH [80] | 1.1% | - | 0.0% | 3.4% | Biracial: 1.1%  Other: 2.3% | 92.1% | - | - | Median education level was a bachelor’s degree | - | - | - | - | Median income was $71K- $80K | 76.1% | 23.9% | - | - |
| YES [78] | - | 3.3% | 6.7% | 3.3% | - | 86.7% | - | - | 100% | - | - | - | - | - | - | - | - | - |

*‘Other’ may include participants in American Indian/Alaska Native and Asian/Pacific Islander categories, as some datasets did not specifically list these racial/ethnic categories.

‘ADTree Model for Axillary Lymph Node Metastasis’ [84, 129], ‘ADTree Model for Pathological Response to Neoadjuvant Therapy’ [84, 130], ‘Breast Cancer Nomograms: Prediction for a Low-risk and a High-risk Oncotype DX Recurrence Score’ [58, 135], ‘Breast Reconstruction Risk Assessment (BRA) Score’ [60, 138], ‘CBCRisk: Contralateral Breast Cancer (CBC) Risk Calculator’ [61, 139], ‘Dynamic Nomogram for Breast IMPC After Mastectomy’ [87, 143], ‘Dynamic Nomogram for Predicting Survival of Locally Advanced Breast Cancer’ [66, 144], ‘LinfoNeo’ [47, 124], Meretoja et. al 2017 [48], ‘METSSS’ [71, 146, 147, 148], ‘Nomogram for Locally Advanced Breast Cancer after Immediate Breast Reconstruction’ [72, 149], ‘Outcomes Predictor after Mastectomy with N1 Breast Cancer’ [44, 125], ‘RSClin’ [13], ‘Stage 4-Breast Cancer Patients’ [86, 155], ‘Surrogate Nomogram for OncotypeDX Recurrence Score’ [83, 156], and ‘Which treatment for DCIS is right for you?’ [46] were validated, but the authors did not provide race, ethnicity, income, education, marital, and insurance status of the population.

‘BreastCHOICE’ [15] and ‘Radiotherapy for Older Women’ [45, 126] were tested for usability/acceptability/feasibility, but the authors did not provide race, ethnicity, income, education, marital, and insurance status information from the population.

FPL: Federal poverty level; NOS: Not otherwise specified; - = None

**Supplemental Table 6. Results from the quality assessment of the web-based treatment and survivorship clinical decision tools based on the International Patient Decision Aids Standards instrument (IPDASi) checklist.**

| **Tool** | **Information about options (0-13)** | **Outcome probabilities (0-9)** | **Clarifying values (0-3)** | **Decision guidance (0-3)** | **Presenting information (0-2)** | **Development process (0-7)** | **Using evidence (0-6)** | **Disclosure and transparency (0-2)** | **Plain language (0-3)** | **Internet-based (0-5)** | **Story usage (0-3)** | **Decision processes (0-6)** | **Decision quality (0-1)** | **Total (0-63)** |
| --- | --- | --- | --- | --- | --- | --- | --- | --- | --- | --- | --- | --- | --- | --- |
| Age Gap Decision Tool – 1 [49, 120] | 6 | 7 | 0 | 1 | 2 | 3 | 3 | 0 | 2 | 5 | 0 | 3 | 0 | **32** |
| Age Gap Decision Tool – 2 [49, 121] | 6 | 7 | 0 | 1 | 2 | 3 | 3 | 0 | 2 | 5 | 0 | 3 | 0 | **32** |
| BreastCHOICE [15] | 9 | 6 | 2 | 3 | 2 | 7 | 3 | 0 | 3 | 4 | 1 | 6 | 0 | **46** |
| BRECONDA [17, 123] | 8 | 0 | 3 | 3 | 1 | 2 | 4 | 1 | 2 | 3 | 1 | 6 | 0 | **34** |
| BTxChoice [14] | 4 | 8 | 0 | 1 | 0 | 1 | 2 | 1 | 1 | 3 | 0 | 0 | 0 | **21** |
| LinfoNeo [47, 124] | 2 | 3 | 0 | 0 | 0 | 2 | 4 | 0 | 2 | 3 | 0 | 2 | 0 | **18** |
| Meretoja et. al 2017 [48] | 1 | 6 | 0 | 0 | 0 | 0 | 0 | 0 | 2 | 2 | 0 | 0 | 0 | **11** |
| Outcomes Predictor after Mastectomy with N1 Breast Cancer [44, 125] | 4 | 8 | 0 | 0 | 1 | 3 | 2 | 0 | 2 | 1 | 0 | 0 | 0 | **21** |
| Radiotherapy for Older Women [45, 126] | 5 | 5 | 0 | 0 | 2 | 4 | 1 | 1 | 3 | 3 | 0 | 2 | 0 | **26** |
| Which treatment for DCIS is right for you? [46] | 7 | 3 | 0 | 2 | 1 | 3 | 1 | 1 | 2 | 3 | 0 | 0 | 0 | **23** |
| 3 Scenarios for Survival [85, 129] | 2 | 3 | 0 | 0 | 0 | 4 | 4 | 1 | 2 | 1 | 0 | 0 | 0 | **17** |
| ADTree Model for Axillary Lymph Node Metastasis [84, 129] | 1 | 4 | 0 | 0 | 0 | 1 | 1 | 0 | 2 | 3 | 0 | 1 | 0 | **13** |
| ADTree Model for Pathological Response to Neoadjuvant Therapy [84, 130] | 1 | 4 | 0 | 0 | 0 | 1 | 1 | 0 | 2 | 3 | 0 | 1 | 0 | **13** |
| ACESO [55] | 3 | 0 | 0 | 2 | 0 | 3 | 0 | 0 | 2 | 4 | 0 | 0 | 0 | **14** |
| Application of Machine Learning Methods to Predict Bone Metastases in Breast Infiltrating Ductal Carcinoma Patients [56, 132] | 2 | 3 | 0 | 0 | 1 | 0 | 0 | 0 | 2 | 3 | 0 | 0 | 0 | **11** |
| Breast Cancer Lung Metastasis Cancer-Specific Survival Nomogram [57, 133] | 2 | 7 | 0 | 0 | 0 | 3 | 0 | 0 | 2 | 2 | 0 | 0 | 0 | **16** |
| Breast Cancer Lung Metastasis Overall Survival Nomogram [57, 134] | 2 | 7 | 0 | 0 | 0 | 3 | 0 | 0 | 2 | 2 | 0 | 0 | 0 | **16** |
| Breast Cancer Nomograms: Prediction for a Low-risk and a High-risk Oncotype DX Recurrence Score [58, 135] | 2 | 4 | 0 | 0 | 0 | 4 | 2 | 0 | 1 | 1 | 0 | 0 | 0 | **14** |
| Breast Cancer Surgery Risk Calculator [59, 137] | 4 | 3 | 0 | 1 | 1 | 3 | 2 | 1 | 2 | 4 | 0 | 0 | 0 | **21** |
| BRA Score [60, 138] | 3 | 3 | 0 | 0 | 1 | 3 | 1 | 0 | 1 | 2 | 0 | 0 | 0 | **14** |
| CBCRisk: Contralateral Breast Cancer (CBC) Risk Calculator [61, 139] | 3 | 5 | 0 | 0 | 0 | 0 | 2 | 0 | 1 | 2 | 0 | 0 | 0 | **13** |
| Conditional Survival Nomogram [62] | 1 | 5 | 0 | 0 | 0 | 0 | 0 | 0 | 2 | 2 | 0 | 0 | 0 | **10** |
| Dai et. al 2018 [63] | 1 | 6 | 0 | 0 | 0 | 0 | 0 | 0 | 2 | 2 | 0 | 0 | 0 | **11** |
| Dynamic Nomogram [64, 141] | 2 | 7 | 0 | 0 | 0 | 4 | 0 | 0 | 2 | 3 | 0 | 0 | 0 | **18** |
| Dynamic Nomogram [65, 142] | 2 | 8 | 0 | 0 | 0 | 0 | 0 | 0 | 2 | 3 | 0 | 0 | 0 | **15** |
| Dynamic Nomogram for Breast IMPC After Mastectomy [87, 143] | 1 | 6 | 0 | 0 | 0 | 0 | 0 | 0 | 2 | 2 | 0 | 0 | 0 | **11** |
| Dynamic Nomogram for Predicting Survival of Locally Advanced Breast Cancer [66, 144] | 0 | 6 | 0 | 0 | 0 | 3 | 1 | 0 | 1 | 2 | 0 | 0 | 0 | **13** |
| Equation [67, 145] | 0 | 3 | 0 | 0 | 0 | 3 | 0 | 0 | 2 | 2 | 0 | 0 | 0 | **10** |
| Huang et. al 2022 [68] | 1 | 4 | 0 | 0 | 0 | 0 | 0 | 0 | 0 | 1 | 0 | 0 | 0 | **6** |
| Huang et. al 2020 [69] (CSS) | 1 | 6 | 0 | 0 | 0 | 0 | 0 | 0 | 2 | 2 | 0 | 0 | 0 | **11** |
| Huang et. al 2020 [69] (OS) | 1 | 6 | 0 | 0 | 0 | 0 | 0 | 0 | 2 | 2 | 0 | 0 | 0 | **11** |
| Li et. al 2021 [70] (CSS) | 1 | 6 | 0 | 0 | 0 | 0 | 0 | 0 | 2 | 2 | 0 | 0 | 0 | **11** |
| Li et. al 2021 [70] (OS) | 1 | 6 | 0 | 0 | 0 | 0 | 0 | 0 | 2 | 2 | 0 | 0 | 0 | **11** |
| METSSS [71, 146] | 3 | 5 | 0 | 0 | 2 | 5 | 5 | 0 | 2 | 4 | 0 | 3 | 0 | **29** |
| Nomogram for Locally Advanced Breast Cancer after Immediate Breast Reconstruction [72, 149] | 2 | 7 | 0 | 0 | 0 | 3 | 0 | 0 | 2 | 1 | 0 | 0 | 0 | **15** |
| Nomogram of Cancer-Specific Survival in Male Breast Cancer Patients with Bone Metastases [73, 150] | 2 | 7 | 0 | 0 | 0 | 0 | 0 | 0 | 2 | 1 | 0 | 0 | 0 | **12** |
| Nomogram of Diagnosis of Bone Metastases in Male Breast Cancer Patients [73, 151] | 2 | 7 | 0 | 0 | 0 | 0 | 0 | 0 | 2 | 1 | 0 | 0 | 0 | **12** |
| Nomogram of Overall Survival in Male Breast Cancer Patients with Bone Metastases [73, 152] | 2 | 7 | 0 | 0 | 0 | 0 | 0 | 0 | 2 | 1 | 0 | 0 | 0 | **12** |
| Nomogram Predicting Survival of Elderly Locally Advanced Breast Cancer [74, 153] | 2 | 7 | 0 | 0 | 0 | 2 | 0 | 0 | 2 | 1 | 0 | 0 | 0 | **14** |
| PersonalRT27 [82, 154] | 1 | 6 | 0 | 0 | 0 | 0 | 0 | 0 | 2 | 2 | 0 | 0 | 0 | **11** |
| Stage 4-Breast Cancer Patients [86, 155] | 1 | 4 | 0 | 0 | 0 | 0 | 2 | 0 | 2 | 2 | 0 | 0 | 0 | **11** |
| Sun et. al 2020 [16] (CSS) | 2 | 6 | 0 | 0 | 0 | 1 | 0 | 0 | 2 | 2 | 0 | 0 | 0 | **13** |
| Sun et. al 2020 [16] (OS) | 2 | 6 | 0 | 0 | 0 | 1 | 0 | 0 | 2 | 2 | 0 | 0 | 0 | **13** |
| Surrogate Nomogram for OncotypeDX Recurrence Score [83, 156] | 1 | 6 | 0 | 0 | 0 | 1 | 2 | 0 | 2 | 2 | 0 | 0 | 0 | **14** |
| Wang et. al 2022 [75] (CSS) | 1 | 6 | 0 | 0 | 0 | 0 | 0 | 0 | 2 | 2 | 0 | 0 | 0 | **11** |
| Wang et. al 2022 [75] (OS) | 1 | 6 | 0 | 0 | 0 | 0 | 0 | 0 | 2 | 2 | 0 | 0 | 0 | **11** |
| Wu et. al 2022 [76] | 1 | 6 | 0 | 0 | 0 | 0 | 0 | 0 | 2 | 2 | 0 | 0 | 0 | **11** |
| Xu et al. 2022 [77] | 1 | 6 | 0 | 0 | 0 | 0 | 0 | 0 | 2 | 2 | 0 | 0 | 0 | **11** |
| Young, Empowered, and Strong (YES) [78] | 0 | 0 | 0 | 2 | 0 | 2 | 0 | 0 | 0 | 4 | 0 | 0 | 0 | **8** |
| Yin et. al 2022 [79] (CSS) | 1 | 6 | 0 | 0 | 0 | 0 | 0 | 0 | 2 | 2 | 0 | 0 | 0 | **11** |
| Yin et. al 2022 [79] (OS) | 1 | 6 | 0 | 0 | 0 | 0 | 0 | 0 | 2 | 2 | 0 | 0 | 0 | **11** |

*’RSClin’ [13], Graetz et. al 2018 [81], and ‘REACH’ [80] were excluded; authors could not accurately assess the full tool.

CSS: cancer-specific survival; OS: overall survival

Color intensity progression is based on the score received in each category with greater intensity signifying a higher score.

Lowest score Highest score

**Information about options:** The tool describes the health condition, lists the options, lists the option of doing nothing, describes the natural course without options, describes procedures, describes positive/negative features, includes the chances of positive/negative outcomes, describes what the test is designed to measure, includes the changes of true positive/true negative/false positive/false negative test results, describes possible next steps based on test results, includes chances the disease is found with/without screening, and describes detection/treatment that would never have caused problems if one wasn’t screened.

**Outcome probabilities:** The tool uses event rates specifying the population and time period, compares outcome probabilities using the same denominator, describes uncertainty around probabilities, uses visual diagrams, uses multiple methods to view probabilities, allows users to select way of viewing probabilities, allows users to view probabilities based on their own situation, places probabilities in context of other events, uses positive and negative frames.

**Clarifying values:** The tool describes procedures and outcomes to help patients imagine what it is like to experience their physical, emotional, and social effects, asks patients to consider which features matter most, and suggests ways for patients to share what matters most with others.

**Decision guidance:** The tool provides steps to decide, suggests ways to talk about the decision with a health professional, and includes tools to discuss options with others.

**Presenting information:** The tool can compare positive/negative features of options and shows negative/positive features with equal detail.

**Development process:** The tool includes developers’ credentials/qualifications, finds out what users need to discuss options, has peer review by patient/professional experts not involved in development and field testing, is field tested with users, is acceptable, is balanced for undecided patients, and is understood by those with limited reading skills.

**Using evidence:** The tool provides references to evidence used, reports steps to find, appraise, and summarize evidence, reports date of last update and how often updated, describes the quality of scientific evidence, and uses evidence from studies of patients similar to those of target audience.

**Disclosure and transparency:** The tool reports sources of funding to develop and distribute the patient decision aid and reports whether authors or their affiliations stand to gain or lose by choices patients make after using the patient decision aid.

**Plain language:** The tool is written at a level that can be understood by the majority of patients in the target group, is written at a grade 8 equivalent level or less according to readability score, and provides ways to help patients understand information other than reading.

**Internet-based:** The tool provides a step-by-step way to move through the web pages, allows patients to search for key words, provides feedback on personal health information that is entered into the patient decision aid, provides security for personal health information entered into the decision aid, makes it easy for patients to return to the decision aid after linking to other web pages, and permits printing as a single document.

**Story usage:** The tool uses stories that represent a range of positive and negative experiences, reports if there was a financial or other reason why patients decided to share their story, and states in an accessible document that the patient gave informed consent to use their stories.

**Decision processes:** The tool helps patients to recognize that a decision needs to be made, know options and their features, understand that values affect decisions, be clear about option features that matter most, discuss values with their practitioner, and become involved in preferred ways.

**Decision quality:** The tool improves the match between the chosen option and the features that matter most to the informed patient [31, 32].
